# Li_21_Ge_8_P_3_S_34_: New Lithium Superionic Conductor with Unprecedented Structural Type

**DOI:** 10.1002/anie.202500732

**Published:** 2025-04-04

**Authors:** Jihun Roh, Saleh Gholam, Namgyu Do, Alicia Manjón‐Sanz, Joke Hadermann, Seung‐Tae Hong

**Affiliations:** ^1^ Department of Energy Science and Engineering DGIST (Daegu Gyeongbuk Institute of Science and Technology) Daegu 42988 Republic of Korea; ^2^ EMAT University of Antwerp Groenenborgerlaan 171 Antwerpen 2020 Belgium; ^3^ Neutron Scattering Division Oak Ridge National Laboratory Oak Ridge TN 37831 USA; ^4^ Department of Chemistry and Chemical Biology University of New Mexico Albuquerque NM 87131 USA; ^5^ NexeriaTek Inc. Daejeon 34016 Republic of Korea

**Keywords:** All‐solid‐state batteries, Conducting materials, Inorganic chemistry, Solid‐state structures, Thio‐LISICONs

## Abstract

Lithium superionic conductors are pivotal for enabling all‐solid‐state batteries, which aim to replace liquid electrolytes and enhance safety. Herein, we report the discovery of an unprecedented lithium superionic conductor, Li_21_Ge_8_P_3_S_34_, featuring a novel structural type and a new composition in the Li–Ge–P–S system. This material exhibits high lithium ionic conductivity of approximately 1.0 mS cm^−1^ at 303 K with a low activation energy of 0.20(1) eV. It's unique crystal structure was elucidated using three‐dimensional electron diffraction (3D ED) and further refined through combined powder X‐ray and neutron diffraction analyses. The structure consists of alternating two‐dimensional slabs: one of corner‐sharing GeS_4_ tetrahedra and the other of isolated PS_4_ tetrahedra, enabling efficient lithium‐ion transport through a tetrahedrally interconnected network of 1D, 2D, and 3D diffusion pathways. This distinctive structural motif provides a novel design strategy for next‐generation solid electrolytes, broadening the structural landscape of lithium superionic conductors. With further advancements in compositional tuning and interfacial engineering, Li_21_Ge_8_P_3_S_34_ could contribute to the development of high‐performance all‐solid‐state batteries.

## Graphical abstract

The discovery of high ion‐conductive inorganic crystal structures remains a challenge in facilitating the development of all‐solid‐state batteries. Ionic conductors reported to date have predominantly been limited to well‐known crystal structures. In this work, we report a new, unprecedented crystalline structure, Li_21_Ge_8_P_3_S_34_, which demonstrates high ionic conductivity.

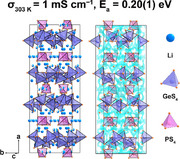

## Introduction

All‐solid‐state batteries (ASSBs) are a promising energy storage solution, offering higher energy density and enhanced safety compared to conventional liquid‐based lithium‐ion batteries, making them ideal for applications such as electric vehicles and grid energy storage systems.^[^ ^1^ ^]^ Lithium superionic conductors are pivotal in enabling ASSBs by replacing liquid electrolytes and addressing associated safety concerns. Over the past few decades, significant progress has been made in developing solid electrolytes for ASSBs.^[^ ^2^, ^3^ ^]^ Notable examples of inorganic lithium‐ion conductors include garnet,^[^ ^4^ ^]^ Li_10_GeP_2_S_12_,^[^ ^5^ ^]^ argyrodite,^[^ ^6^ ^]^ and thio‐LISICONs (LIthium Super Ionic CONductors).^[^ ^7^, ^8^, ^9^ ^]^ These materials exhibit high ionic conductivities, typically ranging from 10^−4^ to 10^−2^ S cm^−1^ at room temperature, highlighting the potential of ASSBs for widespread application.

Despite extensive efforts to develop materials with high ionic conductivities, the diversity of known crystal structures remains limited to a few well‐established families. In recent decades, research has primarily focused on chemically modifying these systems to enhance ionic conductivity and chemical stability,^[^ ^10^, ^11^, ^12^, ^13^, ^14^ ^]^ while discoveries of entirely new structure types have been relatively rare.

The crystal structure framework plays a crucial role in facilitating ion conduction in solid electrolytes. For instance, Li_10_GeP_2_S_12_, discovered by Kanno et al., exhibits one of the highest lithium‐ion conductivities, reaching approximately 10 mS cm^−1^ at room temperature,^[^ ^5^ ^]^ a value comparable to that of conventional liquid electrolytes used in lithium‐ion batteries.^[^ ^15^, ^16^, ^17^ ^]^ Its structure consists of isolated (Ge/P)S_4_ tetrahedra, with lithium ions occupying tetrahedral interstitial sites. These ions form 1D conduction pathways that are interconnected, creating a continuous diffusion network.^[^ ^5^ ^]^ Similarly, the argyrodite structure achieves high ionic conductivity of approximately 10 mS cm^−1^ at room temperature,^[^ ^6^, ^18^, ^19^ ^]^ with lithium ions rapidly diffusing through interconnected cages to form 3D pathways spanning the entire structure.^[^ ^20^ ^]^

Recently, our group reported the crystal structure of Li_2_GeS_3_, composed of infinite chains of corner‐sharing GeS_4_ tetrahedra.^[^ ^21^ ^]^ Although this structure offers sufficient space for lithium‐ion conduction, it exhibits low ionic conductivity (1.63 × 10^−5^ mS cm^−1^ at 303 K), likely due to the lack of lithium‐ion vacancies required for efficient ion hopping. To address this limitation, aliovalent substitution within the structure was explored to create vacancies and enhance conductivity.

Herein, we report a new lithium superionic conductor, Li_21_Ge_8_P_3_S_34_, with a distinct structural type. This compound was discovered through the nominal substitution series Li_2−_ *_x_* Ge_1−_ *_x_* P*_x_* S_3_, designed to introduce vacancies into the parent structure of Li_2_GeS_3_. The Li_21_Ge_8_P_3_S_34_ phase predominates at *x* = 0.25, and its ionic conductivity is significantly enhanced approximately 1.0 mS cm^−1^ at 303 K, with an activation energy of 0.20(1) eV, which is markedly higher than the much lower conductivity of Li_2_GeS_3_ (10^−5^ mS cm^−1^ at 303 K).^[^ ^21^ ^]^ We determined its crystal structure using ab initio structure determination techniques, combining 3D electron, powder X‐ray, and neutron diffraction data.^[^ ^21^, ^22^, ^23^, ^24^, ^25^, ^26^, ^27^, ^28^ ^]^ Bond valence energy landscape (BVEL) calculations based on the resolved crystal structure confirmed its high ionic conductivity. Its electrochemical properties were further evaluated using linear sweep voltammetry (LSV) and galvanostatic discharge/charge measurements in a press cell configuration.

## Results and Discussion

**Figure 1:**
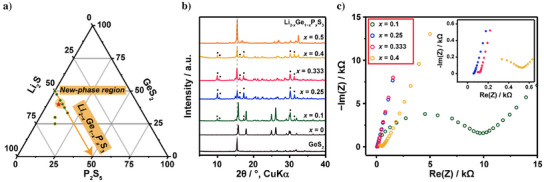
a) Li_2_S−GeS_2_−P_2_S_5_ ternary phase diagram. The orange arrow indicates the nominal substitution series Li_2−x_Ge_1−x_P_x_S_3_, with the red star marking the composition of Li_21_Ge_8_P_3_S_34_. b) Powder XRD patterns of the nominal substitution series Li_2−x_Ge_1−x_P_x_S_3_ for *x* values up to 0.5, synthesized at 793 K for 8 h. Black dots indicate the main peaks corresponding to the Li_21_Ge_8_P_3_S_34_ phase. The XRD pattern of the GeS_2_ precursor is shown at the bottom. c) Nyquist plots of the nominal substitution series Li_2−x_Ge_1−x_P_x_S_3_ for *x* values up to 0.4 at 303 K.

The crystal structure of Li_21_Ge_8_P_3_S_34_ was identified from the nominal substitution series Li_2−_ *_x_* Ge_1−_ *_x_* P*_x_* S_3_, as shown in the ternary phase diagram and X‐ray diffraction (XRD) patterns in Figure 1a,b. XRD patterns along this series revealed the sudden appearance of previously unidentified peaks, which reached maximum intensity at *x* = 0.25 and gradually diminished as the substitution progressed. Simultaneously, peaks corresponding to Li_2_GeS_3_ (*x* = 0) decreased, while those of the GeS_2_ precursor increased. Nyquist plots (Figure 1c) showed a significant reduction in resistance with phosphorous substitution, reaching a minimum resistance and high ionic conductivity (0.92(2) mS cm^−1^ at 303 K) at *x* = 0.25, despite the substantial presence of GeS_2_ and Li_2_GeS_3_. Table S1 lists the ionic conductivity of the Li_2−_ *_x_* Ge_1−_ *_x_* P*_x_* S_3_ substitution series, and Figure S1 shows the fitted Nyquist plots by the equivalent circuit model.

These results suggest that: (i) the Li_2_GeS_3_ crystal structure does not persist throughout the substitution series, (ii) the new phase composition lies between *x* = 0.25 and 0.333, and (iii) this phase is likely to exhibit high ionic conductivity. Motivated by these findings, we conducted exploratory syntheses to isolate the pure phase (see Figure S2) and identify the unknown crystal structure, which exhibited complex powder XRD patterns. Despite testing various synthetic conditions, obtaining a pure phase proved challenging. Consequently, we shifted our focus to solving the crystal structure and determining its precise composition, which then enabled us to synthesize the compound based on the identified structure. Using the three‐dimensional electron diffraction (3D ED) technique,^[^ ^29^, ^30^ ^]^ we acquired 3D ED data on the unknown phase, determined the cell parameters, and solved its crystal structure. Later, we completed the refinement using combined powder X‐ray and neutron diffraction analyses to locate all atoms within the unit cell.

**Figure 2:**
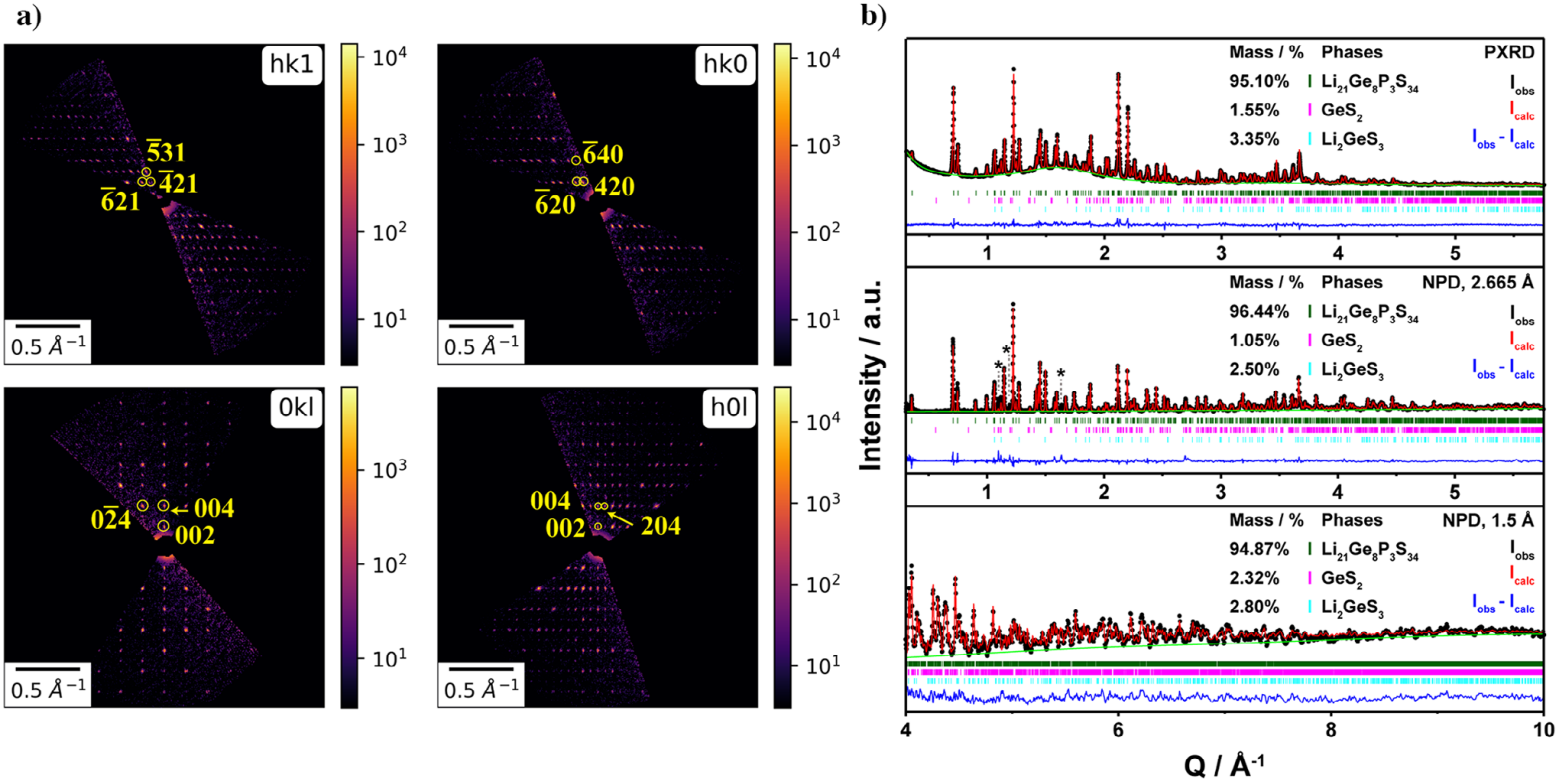
a) Reconstructed 0*kl*, *h*0*l*, *hk*0, and *hk*1 sections from 3D ED data for Li_21_Ge_8_P_3_S_34_ in logarithmic scale. Indexed reflections are highlighted with yellow circles. b) Combined powder X‐ray and neutron Rietveld refinement profiles of Li_21_Ge_8_P_3_S_34_, with minor impurities of GeS_2_ and Li_2_GeS_3_ indicated by pink and cyan Bragg reflection bars, respectively. For the neutron powder diffraction (NPD) data, the different central wavelengths are provided in the upper right corner of each pattern. The asterisk denotes the unidentified phase.

Figure 2a presents a selection of the reconstructed 3D ED patterns from the nineteen 3D ED datasets, specifically showing the 0*kl*, *h*0*l*, *hk*0, and *hk*1 sections of the new phase, Li_21_Ge_8_P_3_S_34_. The unit cell is orthorhombic, with the extinction conditions from these datasets indicating space group *Ccce* (no. 68). Further details are provided in Figures S3–S5. The positions of Ge, P, and S were unambiguously determined based on their distinct electron scattering factors and coordination environments. Charge neutrality considerations allowed us to estimate the lithium content in the unit cell, yielding the chemical composition Li_21_Ge_8_P_3_S_34_. While 3D ED analysis identified 18.6 lithium atoms per formula unit, including partially occupied sites (Figure S6 and Table S2), the remaining lithium atoms could not be resolved. This limitation necessitated further refinement using NPD, which provides enhanced sensitivity to lithium due to its distinct neutron scattering length.^[^ ^31^ ^]^

With the precise chemical composition of the new phase determined, we synthesized the compound according to the formula Li_21_Ge_8_P_3_S_34_. The newly synthesized sample exhibited a reduced amount of GeS_2_​ impurities compared to the nominal series, Li_2−_ *_x_* Ge_1−_ *_x_* P*_x_* S_3_, with *x* = 0.25 and 0.333 (Figure S7). To fully identify the lithium ions in the crystal structure, we conducted NPD experiments on the pure Li_21_Ge_8_P_3_S_34_. Figure 2b shows the Rietveld refinement profiles of combined X‐ray and neutron diffraction patterns for Li_21_Ge_8_P_3_S_34_. All lithium sites were successfully identified with the aid of NPD, which revealed distinct neutron scattering densities in the Fourier maps.

The refined unit cell dimensions are *a* = 35.4145(6) Å, *b* = 11.8907(2) Å, and *c* = 12.6004(2) Å. A broad background peak centered around 1.5 Q (Å^−1^) in the powder XRD patterns is attributed to amorphous carbon intentionally added to the samples to reduce preferred orientation and absorption effects.^[^ ^32^ ^]^ The refined results, including crystallographic data, atomic coordinates, and isotropic thermal parameters, are summarized in Table 1. Selected interatomic distances and angles are provided in Table S3, while bond‐valence sums (BVSs) for each site in Li_21_Ge_8_P_3_S_34_ are listed in Table S4.

**Table 1:** Crystallographic data, Rietveld refinement results, atomic coordinates, site occupancies, and isotropic thermal displacement parameters for Li_21_Ge_8_P_3_S_34_, derived from combined powder X‐ray and time‐of‐flight neutron diffraction analyses.

crystal system	orthorhombic
formula weight (g mol^−1^)	1909.44
space group, Z	*Ccce* (origin choice 2) (no.68), 4
lattice parameters, volume	*a* = 35.4145(6) Å, *b* = 11.8907(2) Å, *c* = 12.6004(2) Å
	*V* = 5306.0(2) Å^3^
density_calc_ (g cm^−3^)	2.39
temperature (K)	298
*R*_wp_/*R*_exp_ (%)^a)^	6.70/2.89
goodness‐of‐fit	2.32
reduced χ^2^	5.38

Atoms	*x*	*y*	*z*	Wyckoff	Occupancy^b)^	*U*_iso_ (Å^2^)
Ge1	0.41182(6)	0.7732(2)	0.6111(2)	16*i*	1.0	0.0303(3)
Ge2	0.37233(7)	0.5379(2)	0.4680(2)	16*i*	1.0	0.0303(3)
S1	0.46238(16)	0.8662(5)	0.6407(5)	16*i*	1.0	0.0345(5)
S2	0.4255(2)	0.6014(5)	0.5494(5)	16*i*	1.0	0.0345(5)
S3	0.3758(3)	0.3538(3)	0.5138(4)	16*i*	1.0	0.0345(5)
S4	0.3756(2)	0.5781(4)	0.3000(4)	16*i*	1.0	0.0345(5)
S5	0.32030(17)	0.5833(5)	0.5381(5)	16*i*	1.0	0.0345(5)
S6	0.3726(2)	0.75	0.75	8*e*	1.0	0.0345(5)
S7	0.28196(17)	0.3508(4)	0.6553(5)	16*i*	1.0	0.0345(5)
S8	0.21680(16)	0.1384(5)	0.6634(5)	16*i*	1.0	0.0345(5)
S9	0.46743(17)	0.6575(4)	0.1533(5)	16*i*	1.0	0.0345(5)
P1	0.24957(14)	0.25	0.75	8*e*	1.0	0.0210(10)
P2	0.5	0.75	0.25	4*a*	1.0	0.0210(10)
Li1	0.9371(4)	0.5035(13)	0.2728(13)	16*i*	1.0	0.065(2)
Li2	0.3354(6)	0.25	0.75	8*e*	0.89(2)	0.065(2)
Li3	0.1700(16)	0.573(5)	0.217(5)	16*i*	0.45(4)	0.065(2)
Li4	0.6916(4)	0.2242(11)	0.4889(11)	16*i*	1.0	0.065(2)
Li5	0.5	0.75	0.75	4*b*	1.0	0.065(2)
Li6	0.0065(9)	0.717(3)	0.001(2)	16*i*	0.471(14)	0.065(2)
Li7	0.0817(8)	0.25	0.25	8*e*	0.82(2)	0.065(2)
Li8	0.25	0	0.518(2)	8*h*	0.96(3)	0.065(2)
Li9	0.75	0.5	0.796(4)	8*h*	0.39(3)	0.065(2)
Li10	0.3207(7)	−0.018(2)	0.764(2)	16*i*	0.55(4)	0.065(2)

^a)^ *R*_wp_ = 100(∑*w*|*I_o_*–*I_c_* |^2^/∑*w*|*I_o_* |^2^)^1/2^; χ^2^ = 100∑*w*|*I_o_*–*I_c_* |^2^/(*N*_obs_‐*N*_var_); *R*_exp_ = *R*_wp_/|χ|.

^b)^ The occupancies of Li1, Li4, and Li5 were fixed at 1.0 due to exceeding the maximum probability during refinement.

To further verify the composition of Li_21_Ge_8_P_3_S_34_, NPD patterns were analyzed for two additional samples with nominal compositions of Li_2−_ *_x_* Ge_1−_ *_x_* P*_x_* S_3_ (*x* = 0.25) and Li_5_GePS_7_, both of which deviate from the Li_21_Ge_8_P_3_S_34_ composition. In both cases, the Li_21_Ge_8_P_3_S_34_ phase consistently appeared as the main phase, albeit with a higher level of impurities compared to the Li_21_Ge_8_P_3_S_34_ sample (Figure S8). These results further supported the accuracy of the determined crystal structure and composition.

**Figure 3:**
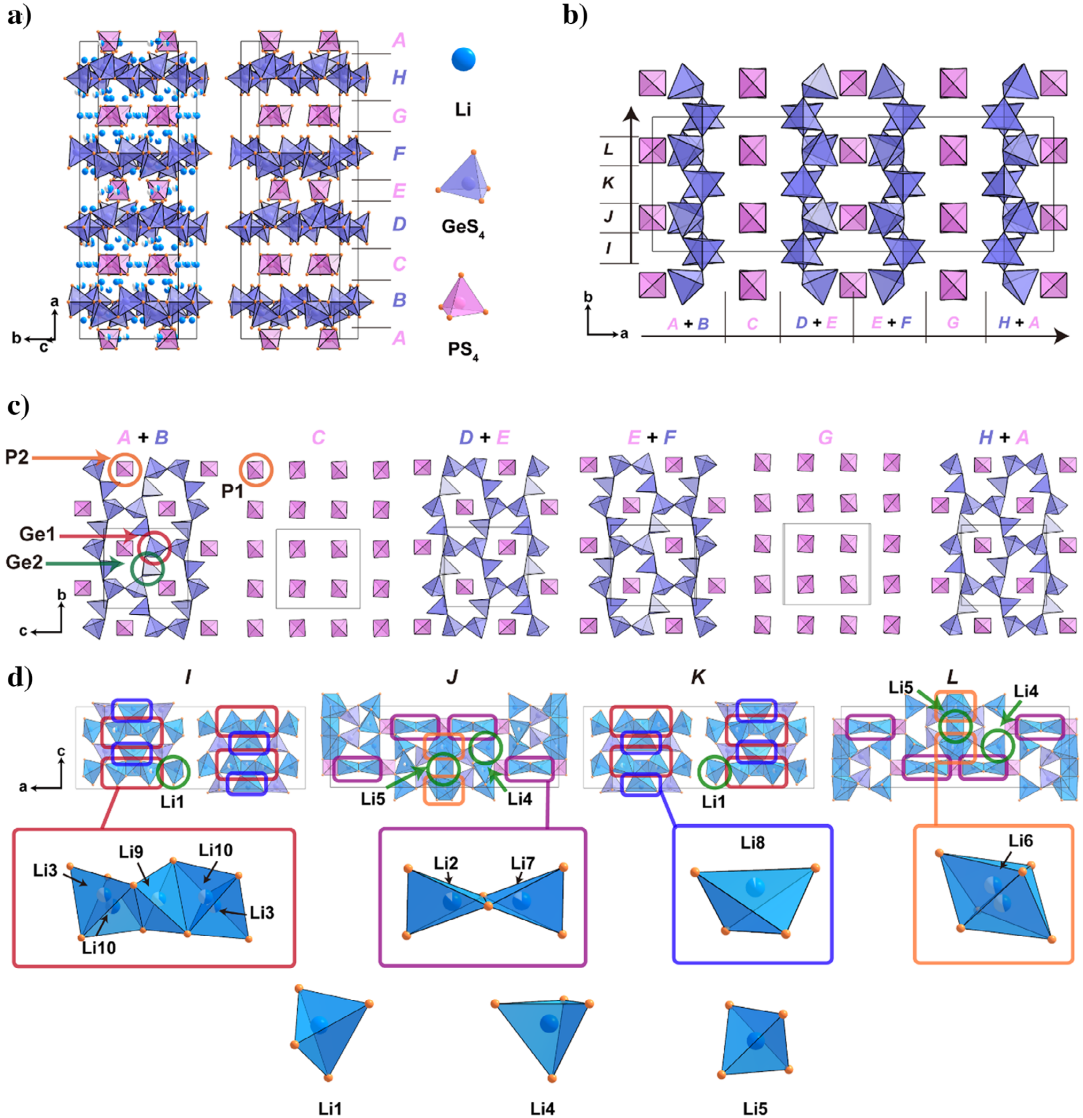
a) Two views of the crystal structure of Li_21_Ge_8_P_3_S_34_, both aligned along the [01$\bar{2}$] direction, showing GeS_4_ and PS_4_​ tetrahedra, with lithium ions represented as spheres. The view on the right omits the lithium ions for clarity. The eight slabs along the *a*‐axis are labeled *A* to *H* and are indicated on the right side of the structure. b) The crystal structure of Li_21_Ge_8_P_3_S_34_ viewed along the [001] direction, showing only the GeS_4_ and PS_4_ tetrahedra for clarity. Slabs are defined along the *a*‐axis (labeled *A* to *H*) and the *b‐*axis (labeled *I* to *L*). c) Slabs *A* to *H* are shown, illustrating the tetrahedral frameworks of GeS_4_ and PS_4_. d) Slabs *I* to *L* are shown, illustrating the arrangement of Li polyhedra, GeS_4_, and PS_4_ tetrahedra. The Li polyhedra are displayed at the bottom, with specific Li polyhedra (Li2, Li3, Li6 to Li10) marked by boxes.

Figure 3a illustrates the crystal structure of Li_21_Ge_8_P_3_S_34_. The unit cell is divided into eight slabs along the *a*‐axis, labeled *A* to *H*, with each slab parallel to the *bc* plane. Slabs *A*, *C*, *E*, and *G* contain isolated PS_4_​ tetrahedra, while Slabs *B*, *D*, *F*, and *H* comprise infinite chains of corner‐sharing GeS_4_​ tetrahedra. These alternating PS_4_ and GeS_4_ slabs repeat along the *a*‐axis. Figure 3b shows four additional slabs dividing the unit cell along the *b*‐axis, labeled *I* to *L*, providing further structural illustration.

Figure 3c details Slabs *A* to *H* along the *a*‐axis. Slabs *B*, *D*, and *F* exhibit infinite sinusoidal 1D chains of corner‐sharing GeS_4_ tetrahedra along the *b*‐axis. Each chain is connected to its neighboring chains by sharing sulfur atoms, creating enclosed voids surrounded by the sinusoidal chains. In the superimposed Slabs (*A* + *B*) and (*H* + *A*), each isolated PS_4_ tetrahedron in Slab *A* is positioned between the voids of the adjacent slabs—situated between the centers of the voids in Slab *B* above and Slab *H* below. Slabs *D*, *E*, and *F* are symmetrically equivalent to Slabs *H*, *A*, and *B*, respectively. Slab *C* consists exclusively of isolated PS_4_ tetrahedra, containing twice as many PS_4_ tetrahedra as Slab *A*. Half of the tetrahedra lie above the void in Slab *B*, and the other half below the void in Slab *D*. Slab *G* is symmetrically equivalent to Slab *C*.

Figure 3d shows Slabs *I* to *L* along the *b*‐axis, parallel to the *ac* plane, illustrating the lithium tetrahedral networks. Slabs *K* and *L* are symmetrically equivalent to Slabs *I* and *J*, respectively. Slab *I* contains a Li3–Li10–Li9–Li10–Li3 tetrahedral connectivity through face‐sharing (Li3–Li10) and edge‐sharing (Li10–Li9), as indicated by the red box. The Li8 tetrahedron, highlighted in the blue box, interconnects these. Slab *J* contains edge‐sharing Li2–Li7 tetrahedra (violet box), a Li6 octahedron (orange box), along with the Li4 and Li5 tetrahedra (green circle). Notably, the Li4 tetrahedron shares corners with two PS_4_ and two GeS_4_ tetrahedra, linking Slabs *C* and *D*, while the Li5 tetrahedron shares corners with four GeS_4_ tetrahedra, linking Slabs *D* and *F*. Additionally, the Li1 tetrahedron shares corners with one PS_4_ and two GeS_4_ tetrahedra, linking Slabs *D* and *E*.

Among the 10 Li sites, only the lithium ions in the slab‐linking tetrahedra (Li1, Li4, and Li5), depicted at the bottom of Figure 3d, are fully occupied, whereas the remaining sites are partially occupied. This structural arrangement indicates that the partially occupied lithium ions primarily contribute to the ionic conductivity in this structure, whereas the fully occupied ions likely play a lesser significant role.

The Li_21_Ge_8_P_3_S_34_ structure shares a feature with Li_2_GeS_3_, where GeS_4_ tetrahedra form 1D chains through corner‐sharing.^[^ ^21^ ^]^ However, Li_21_Ge_8_P_3_S_34_ differs in that these 1D chains are connected to adjacent chains, forming a two‐dimensional corner‐sharing GeS_4_ network, as observed in Slabs *B*, *D*, *F*, and *H*, which are separated by isolated PS_4_ tetrahedra (Slabs *A*, *C*, *E*, and *G*). The distinction in the tetrahedral frameworks between the two crystal structures is illustrated in Figure S9.

In summary, the Li_21_Ge_8_P_3_S_34_ structure comprises alternating two‐dimensional slabs: one consisting of corner‐sharing GeS_4_ tetrahedra and the other of isolated PS_4_ tetrahedra. To the best of our knowledge, Li_21_Ge_8_P_3_S_34_ is the first lithium superionic conductor to exhibit this unique alternation of isolated tetrahedral slabs and corner‐sharing tetrahedral slabs, offering a structural motif that could guide the design of new solid electrolytes.

**Figure 4:**
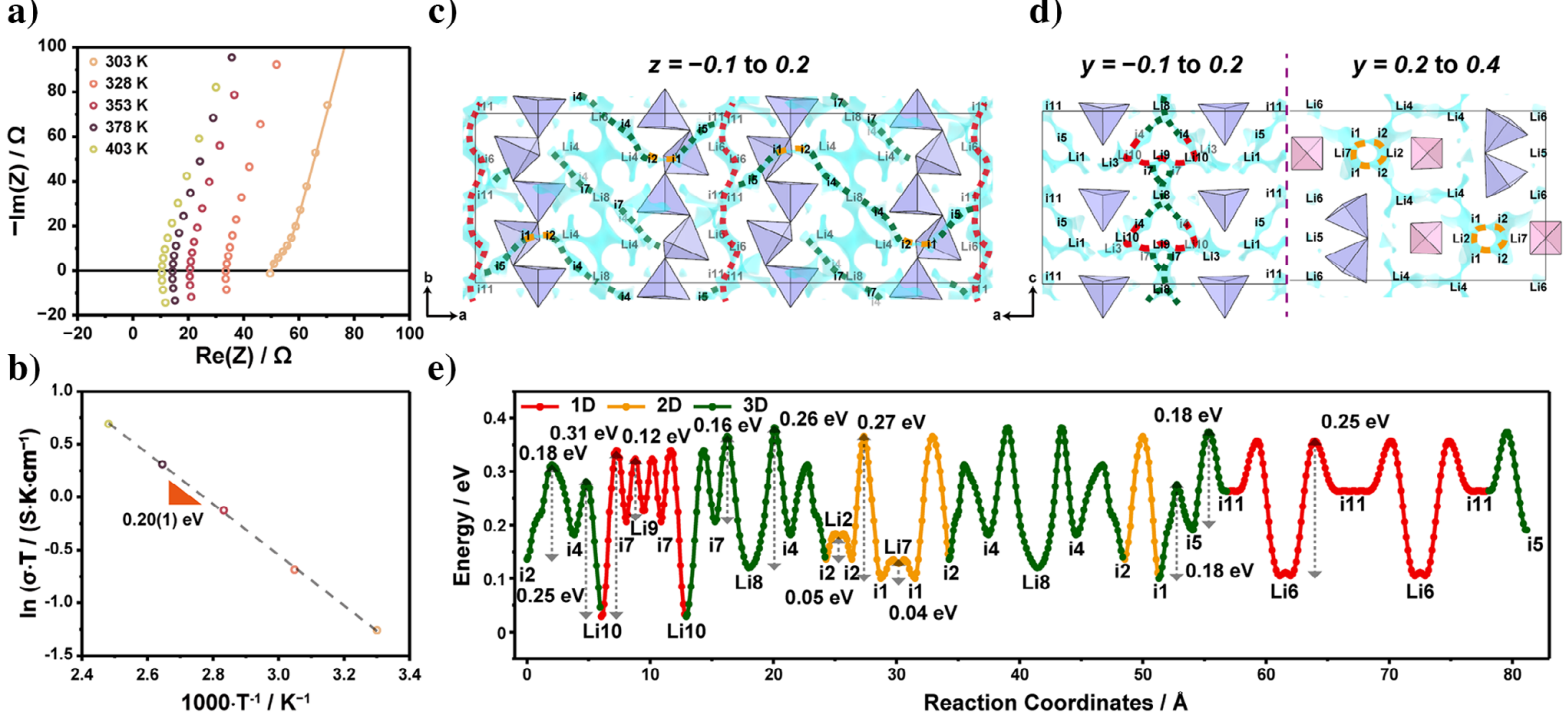
a) Nyquist plots and b) Arrhenius plots of the ionic conductivity of Li_21_Ge_8_P_3_S_34_ measured between 303 and 403 K. The solid light beige line in a) represents the fit using the equivalent circuit model. Lithium‐ion iso‐surfaces are shown in sky‐blue along the c) [001] and d) [010] directions of the crystal structure. For clarity, only a portion of the unit cell is displayed, with a thickness corresponding to the fractional coordinates indicated at the top of the figure. The iso‐surfaces were generated using BVEL calculations, displaying energy levels of 0.4 eV above the global minimum. The dotted lines indicate the lithium‐ion diffusion pathways: 1D (red), 2D (orange), and 3D (green). e) Energy landscape diagram illustrating the lithium‐ion diffusion pathways within the crystal structure.

Figure 4a shows the Nyquist plots of Li_21_Ge_8_P_3_S_34_​ measured at temperatures between 303 and 403 K, revealing the typical impedance response of an ionic conductor. The deconvolution of bulk and grain boundary contributions in resistance is challenging at 328–403 K, likely due to the fast lithium‐ion migration, which is also observed in the literature.^[^ ^10^, ^33^ ^]^ In this case, the equivalent circuit model can be defined as a resistor (R, representing the combined bulk and grain boundary resistance) in series with a constant phase element.^[^ ^33^ ^]^ Therefore, the combined bulk and grain boundary resistances were calculated from the *x*‐intercepts. At 303 K, the Nyquist plot was fitted using the equivalent circuit model, as shown in Figure S10. The overall ionic conductivity, including grain boundary resistance, is 0.95(5) mS cm^−1^, while the bulk ionic conductivity is 1.04(4) mS cm^−1^, comparable to typical values for sulfide‐based ionic conductors (Table 2). Although ∼5% impurities (such as GeS_2_ and Li_2_GeS_3_) were detected in the as‐synthesized Li_21_Ge_8_P_3_S_34_ sample, its ionic conductivity is comparable to that of the *x* = 0.25 composition in the Li_2−_ *_x_* Ge_1−_ *_x_* P*_x_* S_3_ series, which contains 14% impurities and exhibits a conductivity of 0.92(2) mS cm^−1^ (Figure S11 and Table S1). This similarity suggests that the 5% impurity level has a minimal impact on the ionic conductivity of Li_21_Ge_8_P_3_S_34_.

**Table 2:** Comparison of the ionic conductivities of cold‐pressed powders for recently reported superionic conductors, categorized by their original structural types and chemically modified counterparts.

Crystal Structure (type)	Total Ionic Conductivity (mS cm^−1^) at Room Temperature	Activation Energy (eV)	Ref.
Li_21_Ge_8_P_3_S_34_	0.95(5)	0.20(1)	This work
Li_7_P_3_S_11_	3.2	0.12	[34]
Li_10_GeP_2_S_12_ (LGPS)	5, 12 (sintered)	0.25	[5, 35]
LGPS‐Li_9.54_[Si_0.6_Ge_0.4_]_1.74_P_1.44_S_11.1_Br_0.3_O_0.6_	32	0.24	[36]
Li_7_Si_2_S_7_I	10.1(4) (sintered)	0.204(4)	[37]
Li_1.82_SiP_0.036_S_3_	2.4	0.28	[9]
Thio‐LISICON‐* β*‐Li_3_PS_4_	0.16	0.356	[38]
Thio‐LISICON‐* α*‐Li_3_PS_4_	1.3	0.33	[39]
Argyrodite‐Li_6_PS_5_Cl	3.15	0.29	[40]
Argyrodite‐Li_6.5_Sb_0.5_Ge_0.5_S_5_I	16.1	0.18	[41]

The electronic conductivity was measured to be 1.02 × 10^−9^ S cm^−1^, based on the current‐time curves of the stainless steel (SS)/Li_21_Ge_8_P_3_S_34_/SS press cell under 1 V DC polarization (Figure S12). The steady‐state current value was used to calculate the electronic conductivity, as this current is attributed solely to electronic leakage due to the use of ion‐blocking SS electrodes.^[^ ^42^ ^]^ Figure 4b presents the Arrhenius plot of ionic conductivity as a function of temperature, confirming thermally activated conduction with an activation energy of 0.20(1) eV, a sufficiently low value for a lithium superionic conductor. These results demonstrate the promising ionic transport properties of Li_21_Ge_8_P_3_S_34_, which are attributed to its unique crystal structure.

To understand the high ionic conductivity, we conducted lithium‐ion BVEL calculations. Figure 4c,d display the lithium iso‐surfaces in the [001] and [010] directions, respectively, illustrating the diffusion pathways in sky blue. Due to the large unit cell, only a portion is shown for clarity. The diffusion pathways are categorized as 1D (red dotted lines), 2D (orange dotted lines), and 3D (green dotted lines), with the latter interconnecting the 1D and 2D pathways. Figure 4e presents the energy landscape diagram, highlighting the primary diffusion pathways, which exhibit similar energy barriers across the 1D, 2D, and 3D channels.

A favorable 1D lithium‐ion diffusion pathway, [Li6–i11–Li6], with an activation energy of 0.25 eV, runs continuously along the *b*‐axis and is interconnected to 2D and 3D lithium‐ion diffusion channels via the [i5–i1–i2–i4–Li8–i4–i2–i1–i5] network. Another 1D pathway, [Li10–i7–Li9–i7–Li10], is connected to 3D channels via [Li10–i7–Li8–i4–Li10], as shown in Figure 4d. Additional 2D pathways in the *ac* plane, such as [i2–Li2–i2–i1–Li7–i1–i2], are also linked through this 3D channel.

To determine the framework type of Li_21_Ge_8_P_3_S_34_, we applied the polyhedral template matching algorithm,^[^ ^43^ ^]^ a widely used method for identifying framework structures in solids.^[^ ^44^, ^45^, ^46^ ^]^ The analysis revealed that Li_21_Ge_8_P_3_S_34_ does not conform to a single, well‐defined framework type but instead incorporates elements of face‐centered cubic (*fcc*), hexagonal close‐packed (*hcp*), and *bcc* packing motifs (Table S5). This finding suggests that Li_21_Ge_8_P_3_S_34_ cannot be strictly classified within conventional framework categories.

Rather than forming a well‐defined framework, the structure features an alternating arrangement of infinitely corner‐sharing GeS_4_ tetrahedra and isolated PS_4_ tetrahedra, creating well‐connected face‐sharing tetrahedral interstitial sites and forming a continuous percolation network for lithium‐ion migration. As shown in Figure 4e and further illustrated in Figure S13, these interstitial sites provide seamless lithium‐ion diffusion pathways. The calculated positions of lithium, several saddle points, and interstitial sites along the migration pathways, along with their site energies, are listed in Table S6. Direct connectivity between face‐sharing tetrahedral Li sites ensures uniform activation energy barriers, similar to those in other superionic conductors, such as Li_10_GeP_2_S_12_ and Li_6_PS_5_Cl (Figure S14), supporting highly efficient lithium‐ion transport.

Building on this structural connectivity, lithium conduction in Li_21_Ge_8_P_3_S_34_ likely occurs through direct hopping between adjacent face‐sharing tetrahedral or octahedral sites. The low energy barriers associated with these transitions facilitate rapid ionic movement, explaining its high ionic conductivity.

**Figure 5:**
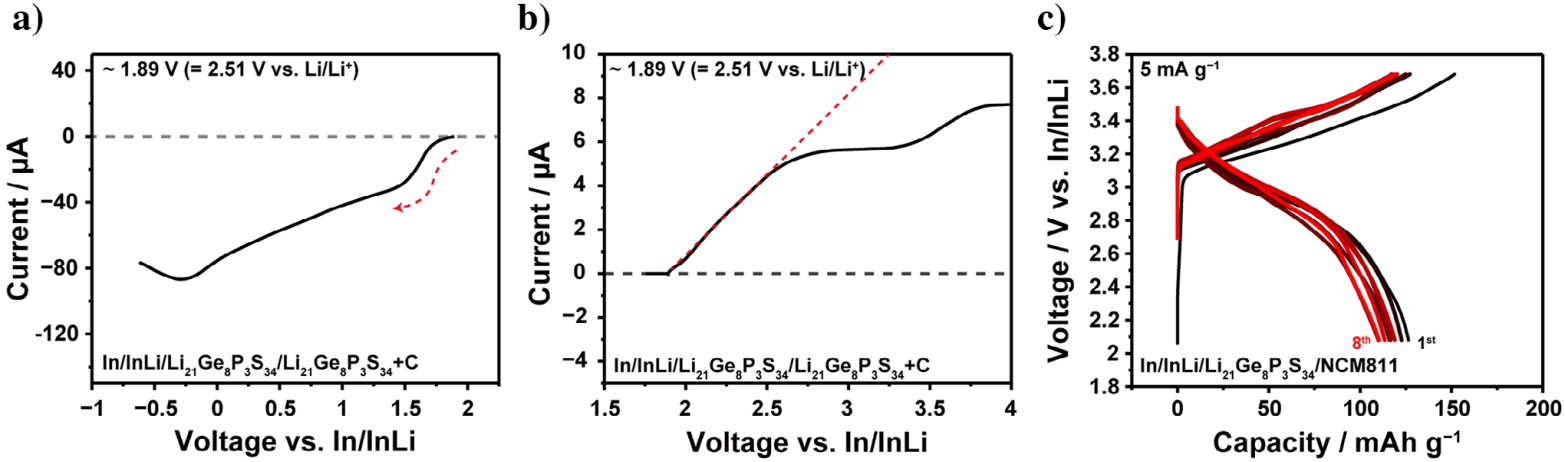
Linear sweep voltammetry (LSV) profile of the press cell configuration In/InLi/Li_21_Ge_8_P_3_S_34_/carbon‐mixed Li_21_Ge_8_P_3_S_34_, performed at a scan rate of 0.05 mV s^−1^ in the voltage range of a) 1.89 to −0.62 V and b) 1.89–4.00 V versus In/InLi. The red dotted line indicates the reduction current curve and onset potential of oxidation. c) Galvanostatic discharge–charge profile of the In/InLi/Li_21_Ge_8_P_3_S_34_/1wt%‐LiNbO_3_ coated LiNi_0.8_Co_0.1_Mn_0.1_O_2_ press cell configuration at a rate of 5 mA g^−1^ over eight cycles.

Figure 5 shows the electrochemical performance of Li_21_Ge_8_P_3_S_34_. Figure 5a,b illustrates the reduction and oxidation stability limits, determined using linear sweep voltammetry (LSV). The intercept of the gray‐dotted lines with the *x*‐axis at 1.89 V versus In/InLi (corresponding to 2.51 V vs. Li/Li^+^)^[^ ^47^ ^]^ marks the onset of oxidation currents, consistent with the previously reported electrochemical stability window of Li_10_GeP_2_S_12_.^[^ ^48^ ^]^ In the reduction voltage region, cathodic currents were significantly greater than anodic currents in the oxidation region, indicating pronounced side reactions during reduction at lower voltages. This suggests that while oxidation occurs at high voltages, reduction‐induced side reactions at lower voltages have a more substantial impact on electrochemical performance.

Figure 5c shows the galvanostatic discharge–charge profile for the press cell configuration In/InLi/Li_21_Ge_8_P_3_S_34_/1wt%‐LiNbO_3_ coated LiNi_0.8_Co_0.1_Mn_0.1_O_2_ (NCM 811) at 5 mA g^−1^. The initial first charge and discharge capacities were approximately 151 and 126 mAh g^−1^, respectively. The discharge and charge capacity remained at ∼110 mAh g^−1^ over eight cycles, suggesting that the oxidation‐induced decomposition of Li_21_Ge_8_P_3_S_34_ has only a modest impact on the cyclability of the interfacial‐coated high‐voltage cathode system.

Additionally, we investigated a low‐voltage cathode press cell configuration, In/InLi/Li_21_Ge_8_P_3_S_34_/TiS_2_. Figure S15 presents its galvanostatic discharge–charge profiles, where the rapid capacity decay observed over eight cycles mirrors the behavior seen in Ge‐containing solid electrolytes.^[^ ^7^, ^49^ ^]^ Since the reduction stability of Li_21_Ge_8_P_3_S_34_ is higher than the operational voltage window (as shown in Figure 5a), this capacity decay is most likely attributed to the reduction‐induced decomposition during cycling.

Overall, Li_21_Ge_8_P_3_S_34_ exhibits promising ionic conductivity, but shares the limited electrochemical stability window common to other superionic conductors, such as Li_10_GeP_2_S_12_.^[^ ^48^ ^]^ However, as demonstrated by the galvanostatic discharge–charge performance of 1wt% LiNbO_3_‐coated NCM 811 (Figure 5c), targeted interfacial engineering and chemical modifications could enhance its electrochemical stability and overall performance.

Beyond its electrochemical properties, Li_21_Ge_8_P_3_S_34_ is a structurally unique lithium superionic conductor, characterized by an unprecedented slab arrangement of isolated and infinitely corner‐sharing tetrahedra. This distinctive structural motif enables lithium‐ion conduction through interconnected diffusion pathways, setting it apart from previously reported superionic conductors. Given the limited diversity of known superionic conductor frameworks, such as Li_10_GeP_2_S_12_ and argyrodite‐type Li_6_PS_5_Cl, this discovery provides a novel structural strategy for designing next‐generation solid electrolytes. Further advancements in compositional tuning and interfacial engineering could improve its electrochemical stability and enhance its performance in all‐solid‐state batteries.

## Conclusions

In this work, we identified Li_21_Ge_8_P_3_S_34_ as a novel lithium superionic conductor with an unprecedented structural type, elucidated through a combination of 3D electron diffraction (3D ED), powder X‐ray, and neutron diffraction analyses. Its distinctive facilitates lithium‐ion diffusion via a tetrahedrally interconnected network of 1D, 2D, and 3D pathways, yielding an ionic conductivity of approximately 1.0 mS cm^−1^ at 303 K and a low activation energy of 0.20(1) eV. Despite its limited electrochemical stability, it exhibits a reversible charge‐discharge profile in an interfacial‐coated high‐voltage cathode press cell configuration.

Beyond its electrochemical properties, the unique slab arrangement of isolated and infinitely corner‐sharing tetrahedra in Li_21_Ge_8_P_3_S_34_ offers fresh insights into lithium‐ion conduction mechanisms. This structural framework not only provides a versatile platform for chemical tuning but also presents new opportunities for exploring structure‐performance relationships in solid electrolytes. Given the limited diversity of known superionic conductor frameworks, such as Li_10_GeP_2_S_12_ and argyrodite‐type Li_6_PS_5_Cl, this discovery expands our understanding of lithium‐ion transport. With further optimization, Li_21_Ge_8_P_3_S_34_ holds significant potential for advancing the design of solid electrolytes in ASSBs.

## Supporting Information

Experimental methods, structural analysis procedures, and supporting figures and tables are given in the Supporting Information. CCDC Deposition Numbers 2408150 contain the supplementary crystallographic data for this paper. These data are provided free of charge by the joint Cambridge Crystallographic Data Centre and Fachinformationszentrum Karlsruhe Access Structures service.

## Conflict of Interests

The authors declare no conflict of interest.

## Supporting information



Supporting Information

## Data Availability

The data that support the findings of this study are available from the corresponding author upon reasonable request.
