# Supplementary material for: Li21Ge8P3S34: New Lithium Superionic Conductor with Unprecedented Structural Type
Source: Angew Chem Int Ed Engl. 2025 Apr 4;64(22):e202500732. doi: 10.1002/anie.202500732 (PMC12105701; doi:10.1002/anie.202500732)
Supplement: Supplementary file 1 — Supporting Information [file ANIE-64-e202500732-s001.pdf]

# Suppporting Information

## $Li_{21}Ge_8P_3S_{34}$: New Lithium Superionic Conductor with Unprecedented Structural Type

*Jihun Roh[a‡], Saleh Gholam[b‡], Namgyu Do[a‡], Alicia Manjón-Sanz[c], Joke Hadermann[b*],*

*Seung-Tae Hong[a, d, e, *]*

[a] Department of Energy Science and Engineering, DGIST (Daegu Gyeongbuk Institute of Science and Technology), Daegu 42988, Republic of Korea

[b] EMAT, University of Antwerp, Groenenborgerlaan 171, 2020 Antwerpen, Belgium

[c] Neutron Scattering Division, Oak Ridge National Laboratory, Oak Ridge, Tennessee 37831, United States

[d] Department of Chemistry and Chemical Biology, University of New Mexico, Albuquerque, New Mexico 87131, United States

[e] NexeriaTek Inc., Daejeon 34016, Republic of Korea

*E-mail: st.hong@dgist.ac.kr

## Experimental Section

***Synthesis:*** All samples were prepared via a solid-state reaction. Stoichiometric amounts of $Li_2S$ (99.9% Metals Basis, Alfa Aesar), $GeS_2$ (99.99%, Kojundo Chemical), and $P_2S_5$ (99%, Sigma-Aldrich) were mixed, ground in an agate mortar, pressed into pellets, and heated at 793 K (with a ramping rate of 2 K $min^{-1}$) for 8 h (for screening purposes) or 20 h in a vacuum-sealed fused-silica ampule. Detailed synthetic conditions and resultant products are provided in the following table.

| Nominal Composition | Heat Treatment Time | Identified phase |
|---|---|---|
| $Li_{2-x}Ge_{1-x}P_xS_3$, $x$ = 0 | 8 h | $Li_2GeS_3$ (ICSD #148058) |
| $Li_{2-x}Ge_{1-x}P_xS_3$, $x$ = 0.1 | 8 h | $Li_2GeS_3$, $Li_{21}Ge_8P_3S_{34}$, $GeS_2$ (ICSD #44) |
| $Li_{2-x}Ge_{1-x}P_xS_3$, $x$ = 0.25 | 8 h, 20 h | $Li_{21}Ge_8P_3S_{34}$, $GeS_2$ |
| $Li_{2-x}Ge_{1-x}P_xS_3$, $x$ = 0.333 | 8 h, 20 h | $Li_{21}Ge_8P_3S_{34}$, $GeS_2$ |
| $Li_{2-x}Ge_{1-x}P_xS_3$, $x$ = 0.4 | 8 h | $Li_{21}Ge_8P_3S_{34}$, $GeS_2$ |
| $Li_{2-x}Ge_{1-x}P_xS_3$, $x$ = 0.5 | 8 h | $Li_{21}Ge_8P_3S_{34}$, $GeS_2$, $Li_4P_2S_6$ (ICSD #434755) |
| $Li_{12}Ge_3P_2S_{17}$ | 8 h | $Li_{21}Ge_8P_3S_{34}$, $Li_2GeS_3$ |
| $Li_5GePS_7$ | 8 h, 20 h | $Li_{21}Ge_8P_3S_{34}$, $Li_2GeS_3$, $Li_{3.1}Ge_{0.1}P_{0.9}S_4$[a] |
| $Li_{21}Ge_8P_3S_{34}$ | 20 h | $Li_{21}Ge_8P_3S_{34}$, $Li_2GeS_3$, $GeS_2$ |

[a]Phase identified through Rietveld refinement of neutron powder diffraction data, as shown in **Figure S7**, previously reported in ref.[1]

***Powder X-ray diffraction with Bragg–Brentano geometry:*** Powder X-ray diffraction (XRD) measurements were performed using a Rigaku MiniFlex 600 diffractometer in Bragg–Brentano geometry, employing Cu Kα radiation (λ = 1.5418 Å) and a secondary graphite (002) monochromator. Data were collected over a scanning range of 5° ≤ 2θ ≤ 40°, with a step size of 0.02°, to evaluate the phase purity of the synthesized samples. The samples were prepared in a glovebox to prevent air exposure and sealed in an airtight XRD sample holder with a Kapton

polyimide film (Rigaku).

***3D electron diffraction:*** Three-dimensional electron diffraction (3D ED) experiments were conducted on a Thermo Fischer Scientific Tecnai Osiris transmission electron microscope (TEM) operating at 200 kV, equipped with a US1000 XP CCD. A small amount of the sample ($x$ = 0.333 in $Li_{2-x}Ge_{1-x}P_xS_3$) was crushed between two microscope slides inside a glove box. Particles were collected by rubbing a copper grid with an ultra-thin continuous carbon support (Electron Microscopy Sciences) on the slides. A GATAN vacuum transfer holder was used to prevent air exposure during experiments.

To minimize beam damage, a minimal electron dose was applied using a 10-µm C2 aperture and a high spot size. Dose and dose rate were calculated from detector calibration and particle images. Data acquisition involved continuously tilting the holder at 0.517°/s from −35° to 32° using an in-house script. Each frame, with 1 fps exposure time, integrated 0.517° of reciprocal space accounting for 0.014° lost due to the detector read-out.

After tomography, a 50-µm C2 aperture and a low spot size were used to increase the electron dose for energy-dispersive X-ray spectroscopy (EDS) measurements using a Super-X detector. EDS quantification, performed with TEM Imaging and Analysis software (Thermo Fischer Scientific), confirmed particle composition and ruled out impurities.

***Powder X-ray diffraction with Debye–Scherrer geometry:*** To obtain higher-quality data for the ab-initio structure determination of $Li_{21}Ge_8P_3S_{34}$, we conducted powder XRD measurements using an X-ray diffractometer (Bruker-AXS D8 Advance) in Debye–Scherrer geometry with Cu $K\alpha_1$ ($\lambda$ = 1.5406 Å, 40 kV, and 45 mA) radiation, a focusing primary Ge (111) monochromator, and a Vantec position-sensitive detector with a detector slit of 6°. The powder samples were mixed with

carbon (super C, TIMCAL) at a 2:1 weight ratio to ensure homogeneous distribution, reduce preferred orientation effects, lower packing density, and mitigate absorption effects. The sample was placed in a glass capillary with a diameter of 0.05 cm, which was sealed with wax and double-sealed with vacuum grease to prevent air exposure. Measurements were taken over an angular range of $3° \leq 2\theta \leq 130°$ with a step size of 0.0166903°, a total measurement time of 12 h at room temperature.

***Neutron powder diffraction:*** A time-of-flight (TOF) NPD experiment was conducted using the POWGEN diffractometer (BL-11A beamline) at the Spallation Neutron Source (SNS) at Oak Ridge National Laboratory.[2] Each powder sample (nominal composition of $x$ = 0.25 in $Li_{2-x}Ge_{1-x}P_xS_3$, $Li_5GePS_7$, and $Li_{21}Ge_8P_3S_{34}$) was loaded in a cylindrical vanadium can ($\phi$ = 0.6 cm) and sealed with a copper gasket in an Ar-filled glovebox to prevent air exposure. Central wavelengths of 1.5 Å and 2.665 Å were used to cover *d*-spacing ranges of 0.5–11.8 Å and 1.1–20.5 Å, respectively. Data collection times were adjusted based on sample weight: 1.5 h for $x$ = 0.25 in $Li_{2-x}Ge_{1-x}P_xS_3$, 3 h for $Li_5GePS_7$, and 2 h for $Li_{21}Ge_8P_3S_{34}$. All measurements were performed at room temperature.

***Ab initio structure determination:*** For the ab initio structure determination of $Li_{21}Ge_8P_3S_{34}$ using 3D ED, PETS2 was used for the data reduction and reciprocal space section creation,[3] SHELXT for the structure solution, and Jana2020 for both kinematical and dynamical refinement.[4,5] A total of 32 datasets from 29 different particles were acquired in a single microscope session. Out of these, 10 datasets were discarded due to the presence of multiple domains and mismatched Ge/P ratios (as measured using energy dispersive X-ray analysis) relative to the nominal composition ($x$ = 0.25 or 0.333) in $Li_{2-x}Ge_{1-x}P_xS_3$. The remaining datasets exhibited the same unit cell, with minor variations arising from typical distortions in 3D ED data.[6] These distortions were refined with

reference to the cell parameters refined with neutron powder diffraction data.[7] Two datasets, corresponding to particles P1 and P2 (**Figure S16**), are mainly discussed here. The structure solution and refinement were performed on P1, and the results are presented in **Table S2**, **S7–8**.

The structure refined from 3D ED served as the basis for extracting structure factors from powder XRD data through Le Bail fitting, conducted using the powder refinement software GSAS II.[8] Refinement parameters at this stage included the peak shape profile function, background function, and unit cell parameters. The extracted structure factors were subsequently input into the single-crystal refinement program, CRYSTALS.[9]

A structural model was reconstructed from scratch using a charge-flipping algorithm in SUPERFLIP within the CRYSTALS suite.[10] The atomic positions of Ge, P, and S—elements with relatively high atomic numbers—were identified through Fourier electron density map visualization with MCE. These closely matched the structural model determined by 3D ED,[11] validating the structural solution. However, Li positions remained ambiguous based on XRD data alone. Neutron diffraction data was subsequently employed to identify the Li positions. Final refinement of atomic positions and isotropic thermal displacements was performed using combined X-ray and neutron Rietveld refinement in GSAS II.[8]

BVS calculations, performed using softBV (V.1.2.7), yielded reasonable values for each atomic site,[12] further supporting the structural accuracy (**Table S4**). BVEL calculations were also conducted using softBV (V.1.2.7) with a resolution of 0.1 Å. The crystal structure and Li-ion energy landscapes were visualized using VESTA.[13]

The sulfur sublattice was analyzed using the polyhedral template matching algorithm within the software Ovito.[14,15]

***Electrochemical impedance and DC polarization analysis:*** The ionic conductivity of $Li_{21}Ge_8P_3S_{34}$ was determined using temperature-dependent electrochemical impedance spectroscopy (EIS). Measurements were conducted with a custom-built press cell and a Biologic SP-200 impedance analyzer (Biologic Science Instruments) over a temperature range of 303–403 K and a frequency range of 2 MHz to 1 Hz, with an applied amplitude of 55 mV. The nominal substitution series in $Li_{2-x}Ge_{1-x}P_xS_3$ ($0.1 \leq x \leq 0.5$) were analyzed using the same method, except for a different frequency range of 1 MHz to 1 Hz. $Li_{21}Ge_8P_3S_{34}$ powder was placed between two lithium-ion blocking Ti electrodes within a poly(etheretherketone) (PEEK) cylinder, and a pressure of 375 MPa was applied using a hydraulic press. After EIS measurements, the thickness of the pressed sample was measured to be approximately 0.041(2) cm using a vernier caliper after disassembling the cell. A heating block was used to regulate the temperature during measurement. In contrast, the aliovalent substitution series $Li_{2-x}Ge_{1-x}P_xS_3$ ($x$ = 0.1, 0.25, 0.333, 0.4, and 0.5) was characterized only at 303 K under identical conditions to $Li_{21}Ge_8P_3S_{34}$. The electronic conductivity of $Li_{21}Ge_8P_3S_{34}$ was determined using a press cell configuration of SS/$Li_{21}Ge_8P_3S_{34}$/SS under 1 V DC polarization.

***Linear sweep voltammetry analysis:*** Linear sweep voltammetry (LSV) was conducted using a press cell configuration (**Figure S17**) consisting of the components: In/InLi/$Li_{21}Ge_8P_3S_{34}$/carbon-mixed $Li_{21}Ge_8P_3S_{34}$. A 150 mg pellet of $Li_{21}Ge_8P_3S_{34}$ powder was pressed under a pressure of 375 MPa. Subsequently, 20 mg of carbon-mixed $Li_{21}Ge_8P_3S_{34}$ powder (prepared at a 7:3 sample-to-carbon weight ratio) was evenly spread on one side of the pellet. On the other side, In/InLi foil (ϕ = 1.1 cm; Li 1.5 wt%, In 98.5 wt%, NEBA) was placed. The assembled cell was secured with an alloy screw nut tightened to a torque of 8.04 Nm. After stabilizing the open-circuit voltage, a linear

voltage sweep was applied up to 4 V vs. In/InLi and −0.62 V vs. In/InLi at a scan rate of 0.05 mV $s^{-1}$ at room temperature.

***Galvanostatic discharge/charge analysis:*** Galvanostatic discharge–charge cycle measurements were conducted using a press cell configuration with an In/InLi alloy anode, a $Li_{21}Ge_8P_3S_{34}$ solid electrolyte, and 1wt%-$LiNbO_3$ coated $LiNi_{0.8}Co_{0.1}Mn_{0.1}O_2$ (NCM811, L&F) or $TiS_2$ composite cathode, all assembled in a glovebox. Prior to use, $TiS_2$ powder was ball-milled at 600 rpm for 8 h. The 1wt%-$LiNbO_3$ coated NCM811 cathode composite was prepared by mixing 1wt%-$LiNbO_3$ coated NCM811 and $Li_{21}Ge_8P_3S_{34}$ in a 70:30 weight ratio using a planetary centrifugal mixer (ARE-310, Thinky) at 2000 rpm for 15 minutes, with three 3 mm $ZrO_2$ balls in an ointment container. The $TiS_2$ cathode composite was prepared by mixing $TiS_2$ and $Li_{21}Ge_8P_3S_{34}$ powders in a 1:2 weight ratio using an agate mortar and pestle. For cell assembly, 150 mg of $Li_{21}Ge_8P_3S_{34}$ powder was pressed into a pellet with a diameter of 1.3 cm under 375 MPa pressure. The cathode composite powder was evenly spread on one side of the solid electrolyte pellet, while the In/InLi alloy anode was placed on the opposite side. The cell was secured using an alloy screw nut tightened to a torque of 8.04 N·m, corresponding to an approximate pressure of 60 MPa. The cell was cycled within a voltage range of 2.08–3.68 V vs. In/InLi for 1wt%-$LiNbO_3$ coated NCM811 cathode composite, and 0.78–2.08 V vs. In/InLi for $TiS_2$ cathode composite. The discharge–charge process was conducted at a constant current of 5 mA $g^{-1}$ for 1wt%-$LiNbO_3$ coated NCM811 cathode composite, and 23.9 mA $g^{-1}$ for $TiS_2$ cathode composite up to 8 cycles in this solid-state battery system.

## Supporting Figures and Tables

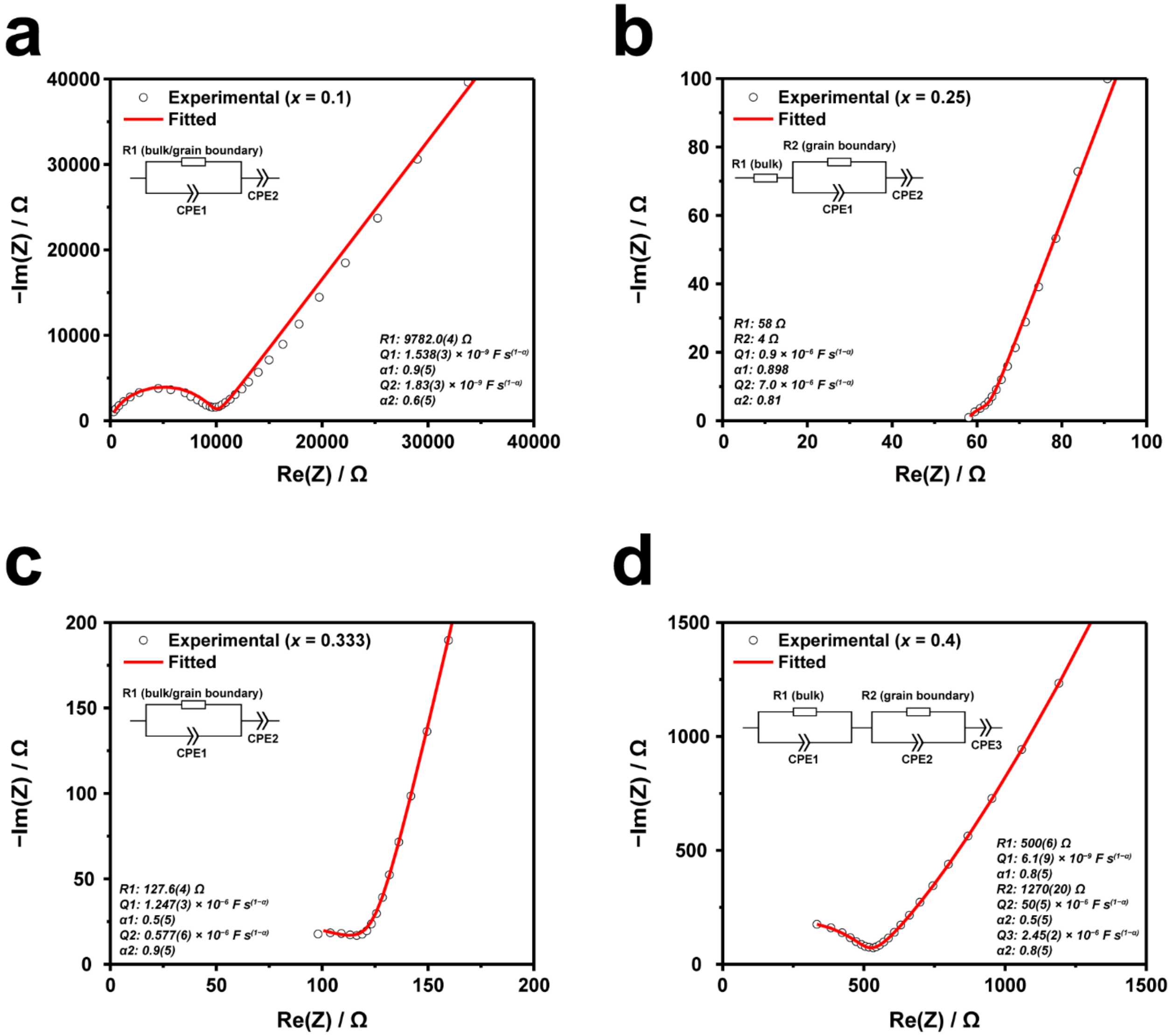


**Figure S1**. Nyquist plots for the nominal substitution series of $Li_{2-x}Ge_{1-x}P_xS_3$ (heat-treated for 8 h) at 303 K: (a) $x$ = 0.1, (b) $x$ = 0.25, (c) $x$ = 0.333, (d) $x$ = 0.4. Insets in each figure depict the corresponding equivalent circuit model, with circuit component values indicated at the bottom of the respective figures. Solid red lines represent the fitted curves, while experimental data points are shown as circles. The ionic conductivities for each composition are listed in **Table S1**.

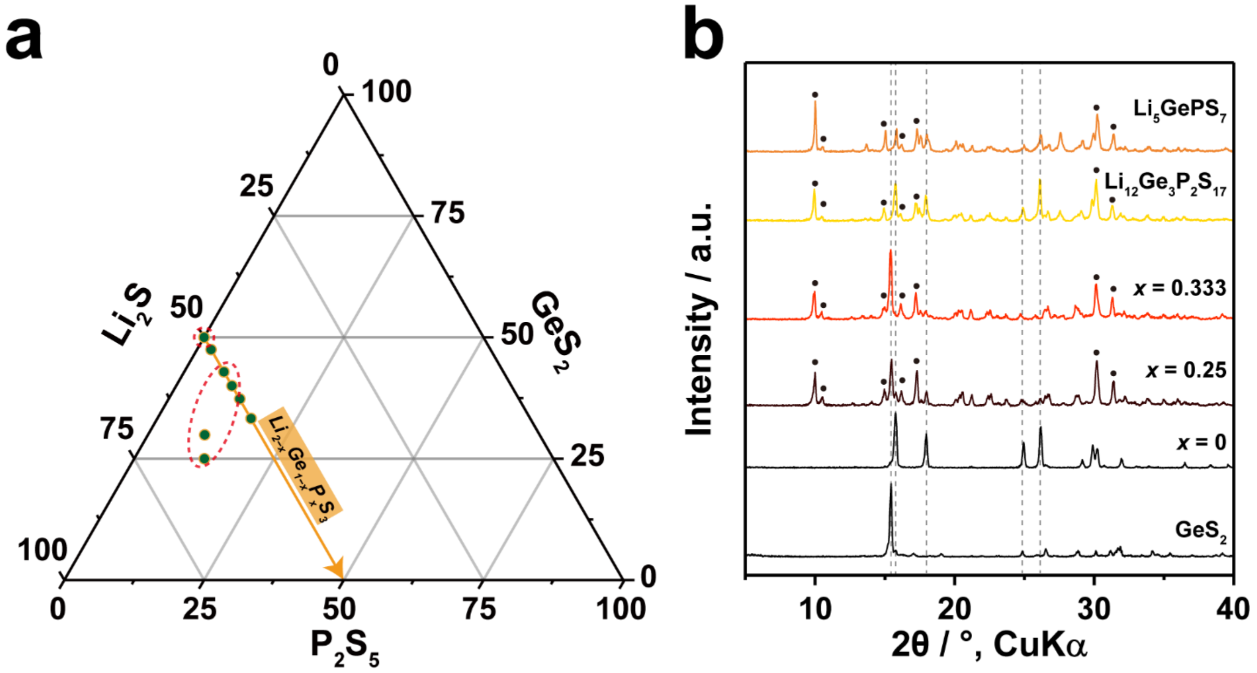


**Figure S2**. (a) Ternary phase diagram of $Li_2S$–$GeS_2$–$P_2S_5$, highlighting the nominal compositions of $x$ = 0.25 and 0.333 in $Li_{2-x}Ge_{1-x}P_xS_3$, $Li_{12}Ge_3P_2S_{17}$, and $Li_5GePS_7$ phases (marked by a red dotted circle). (b) Powder XRD patterns for these nominal compositions, synthesized at 793 K for 8 h. Gray dotted lines indicate peaks associated with $Li_2GeS_3$ and $GeS_2$, while black circles denote main peaks corresponding to the $Li_{21}Ge_8P_3S_{34}$ phase.

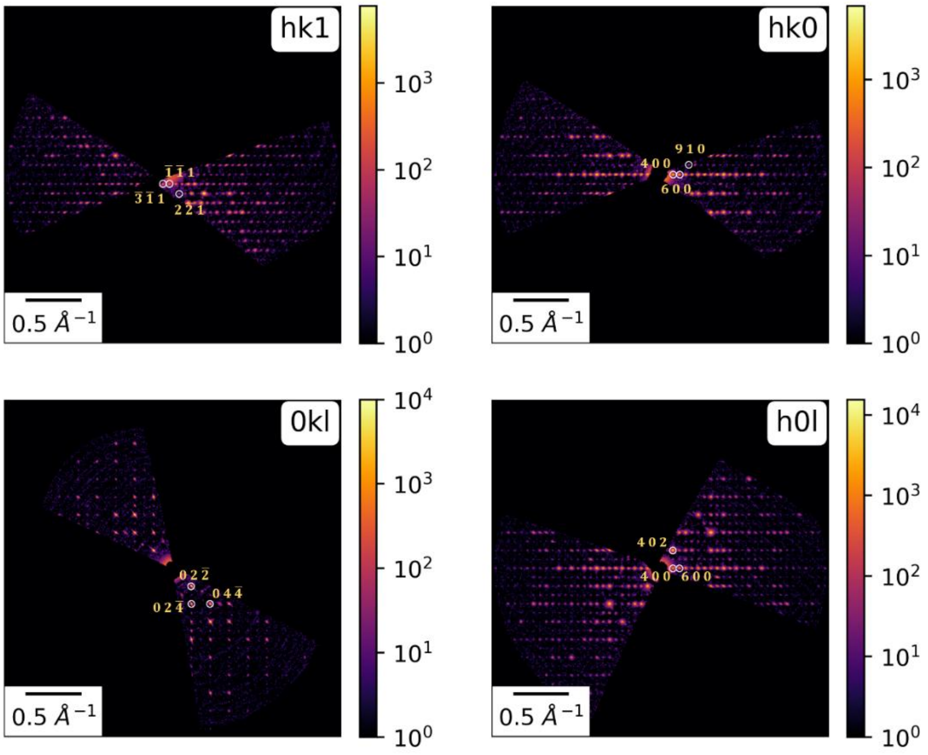


**Figure S3.** Reciprocal space sections for P2 on a logarithmic scale.

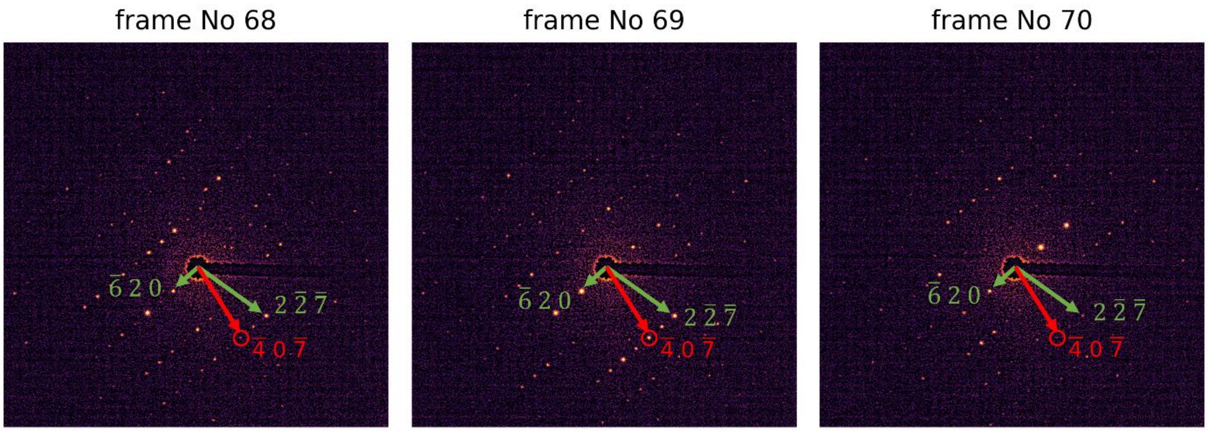


**Figure S4.** The possible dynamical scattering path responsible for the appearance of the $\bar{4}0\bar{7}$ reflection in P1.

**Figure 2a** and **Figure S3** present the reconstructed reciprocal space sections for particles P1 and P2, respectively. Based on these sections, the following reflection conditions can be derived: *hkl:* $h+k = 2n$, *hk*0*:* $h+k = 2n$, 0*kl:* $k = 2n$, *h*0*l:* $h = 2n$. These reflection conditions lead to the space groups *C*222, *Cmm*2, *Cm*2*m*, *C*2*mm,* or *Cmmm*. Nevertheless, a detailed inspection reveals that the 0*kl:* $l = 2n+1$ and *h*0*l:* $l = 2n+1$ reflections are much weaker than the 0*kl:* $l = 2n$ and *h*0*l:* $l =$

$2n$ reflections. Similarly, the $hk0$: $h = 2n+1$ and $hk0$: $k = 2n+1$ reflections are much weaker than the $hk0$: $h, k = 2n$ reflections. This indicates the possibility of dynamic scattering effects causing such violations. For example, if $h0l$: $h, l = 2n$ applies instead of $h0l$: $h = 2n$, then the $\bar{4}\ 0\ \bar{7}$ reflection should be extinct for P1. Its appearance on the $h0l$ section can be traced back to two frames shown in **Figure S4**, where the $\bar{4}0\bar{7}$ reflection is the vector addition of two strong reflections seen in that frame (and can thus be the result of double diffraction). Similar origins can be found for the other violating reflections. Taking these intensity differences into consideration as being systematic extinctions, the space group would be either *Ccc*2 or *Ccce*.

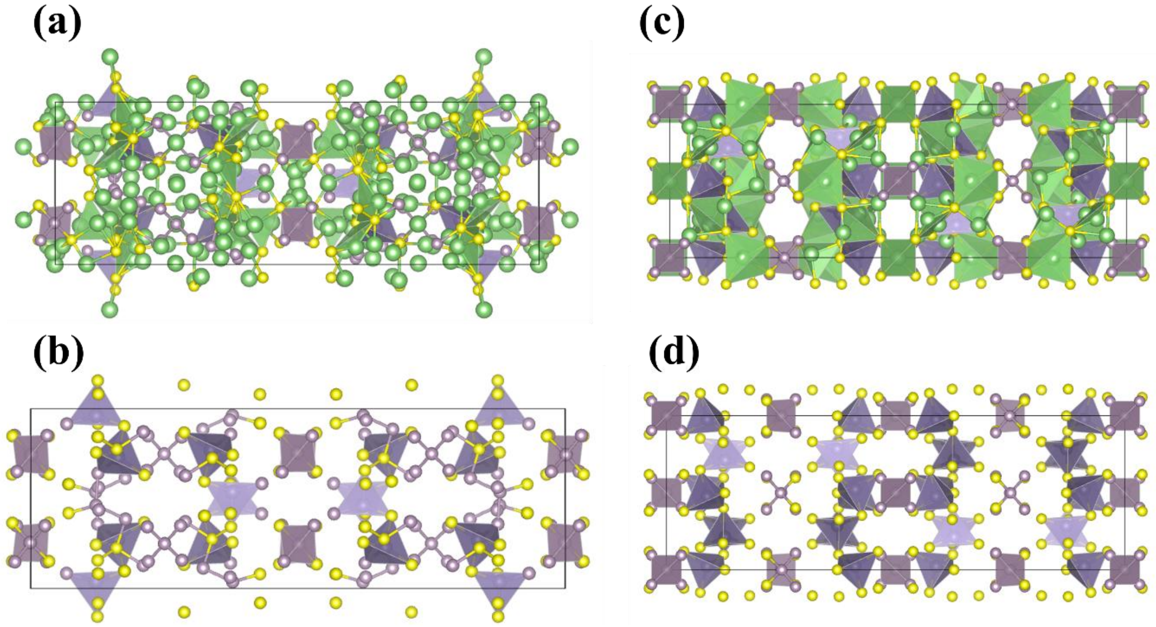


**Figure S5**. The initial structure model for P1, generated using SHELXT. (a) Space group *Ccc*2; (b) *Ccc*2 without Li atoms; (c) Space group *Ccce*; (d) *Ccce* without Li atoms. Atoms are represented as follows: Li (green), Ge (deep violet), and P (light violet).

Different structure solution algorithms were tested on multiple datasets. SUPERFLIP always converged to space group *Ccce*, although the structure did not make sense chemically as it had a lot of atoms close to each other[10]. SHELXT solutions were of much higher quality and the solutions for both space groups *Ccc*2 and *Ccce* seemed reasonable (**Figure S5**).[4] In the case of space group *Ccce*, most of the atoms in the polyhedra seemed to be assigned correctly, including several Li atoms. For structure solution by SIR2019, it is possible to impose a space group on the solution algorithm, and *Ccc*2, *C*222, and *Ccce* had the best solutions, however, structure refinement in *C*222 failed[16]. Thus, it was clear that either *Ccce* or *Ccc*2 should be the space group, and the violations in the sections were indeed induced by dynamical scattering.

Because of their higher quality, the structures solved by SHELXT were used for structure refinement. After the kinematical refinement, the structure based on *Ccc*2 showed several weaknesses. One Ge atom, one S atom, and one Li atom showed negative atomic displacement

parameters ($U_{iso}$). Dynamical refinement improved $U_{iso}$ for Ge and S atoms, but the $U_{iso}$ for several other atoms decreased to unrealistically low values. None of these issues appeared in the case of the *Ccce* space group. Five Li atoms were found in this structure during kinematical refinement, and all of them had positive $U_{iso}$. These results indicate that the structure should have space group *Ccce*.

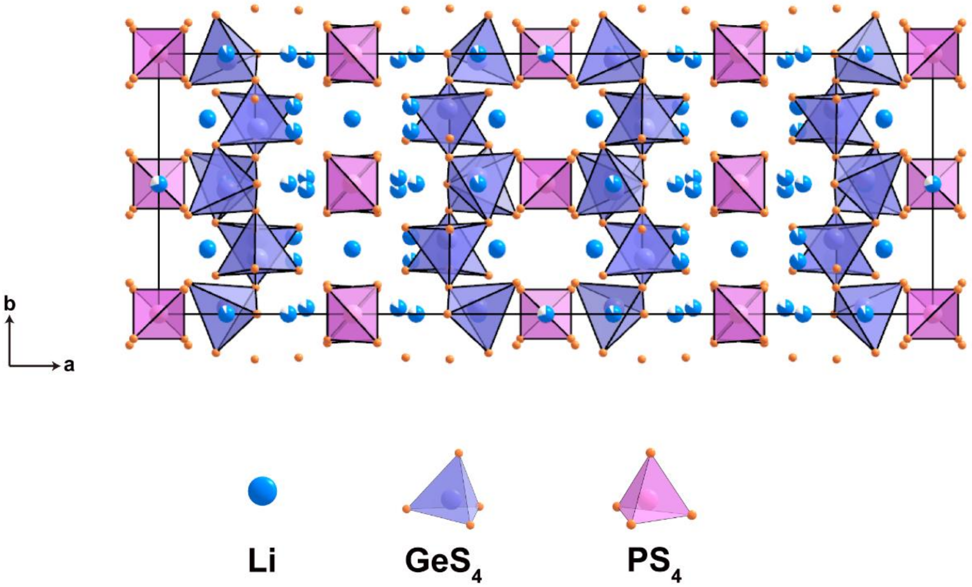


**Figure S6**. The crystal structure of $Li_{18.6}Ge_8P_3S_{34}$, with lithium positions not fully resolved, as determined using 3D ED.

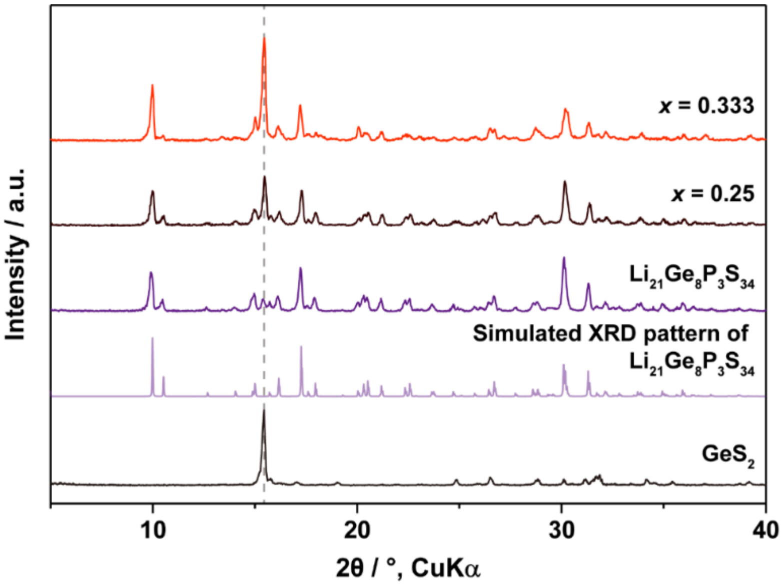


**Figure S7**. Powder XRD patterns of $x = 0.25$ and 0.333 in $Li_{2-x}Ge_{1-x}P_xS_3$, along with $Li_{21}Ge_8P_3S_{34}$, synthesized at 793 K for 20 h. The gray dotted lines indicate peaks corresponding to $GeS_2$. The simulated XRD pattern for $Li_{21}Ge_8P_3S_{34}$ is shown in light violet.

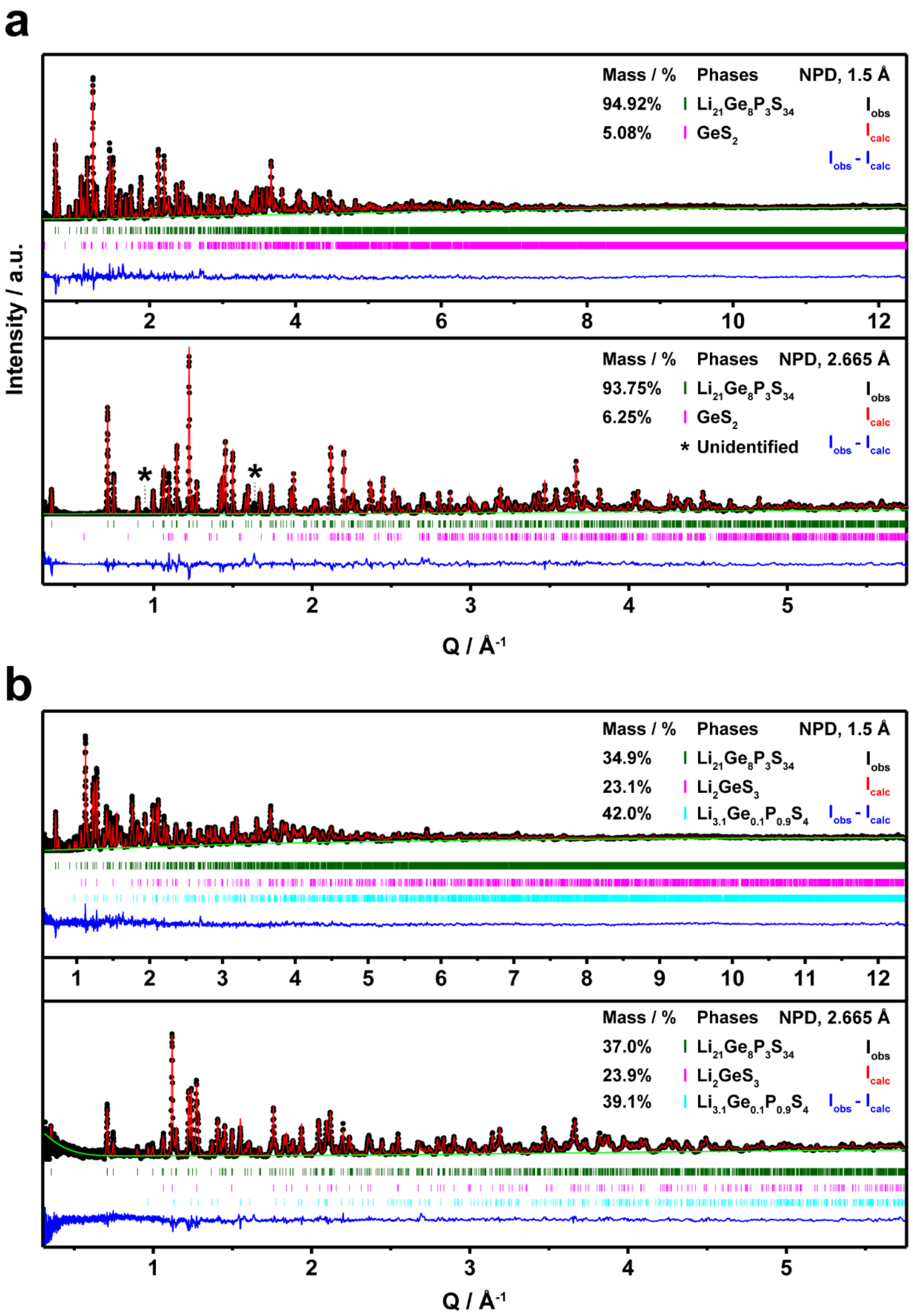


**Figure S8**. Combined powder neutron Rietveld refinement (using central wavelengths of 1.5 Å and 2.665 Å) for the nominal compositions of (a) $x = 0.25$ in $Li_{2-x}Ge_{1-x}P_xS_3$ and (b) $Li_5GePS_7$, synthesized at 793 K for 20 h. The mass fraction is indicated in the upper right corner. The asterisk denotes the unidentified phase.

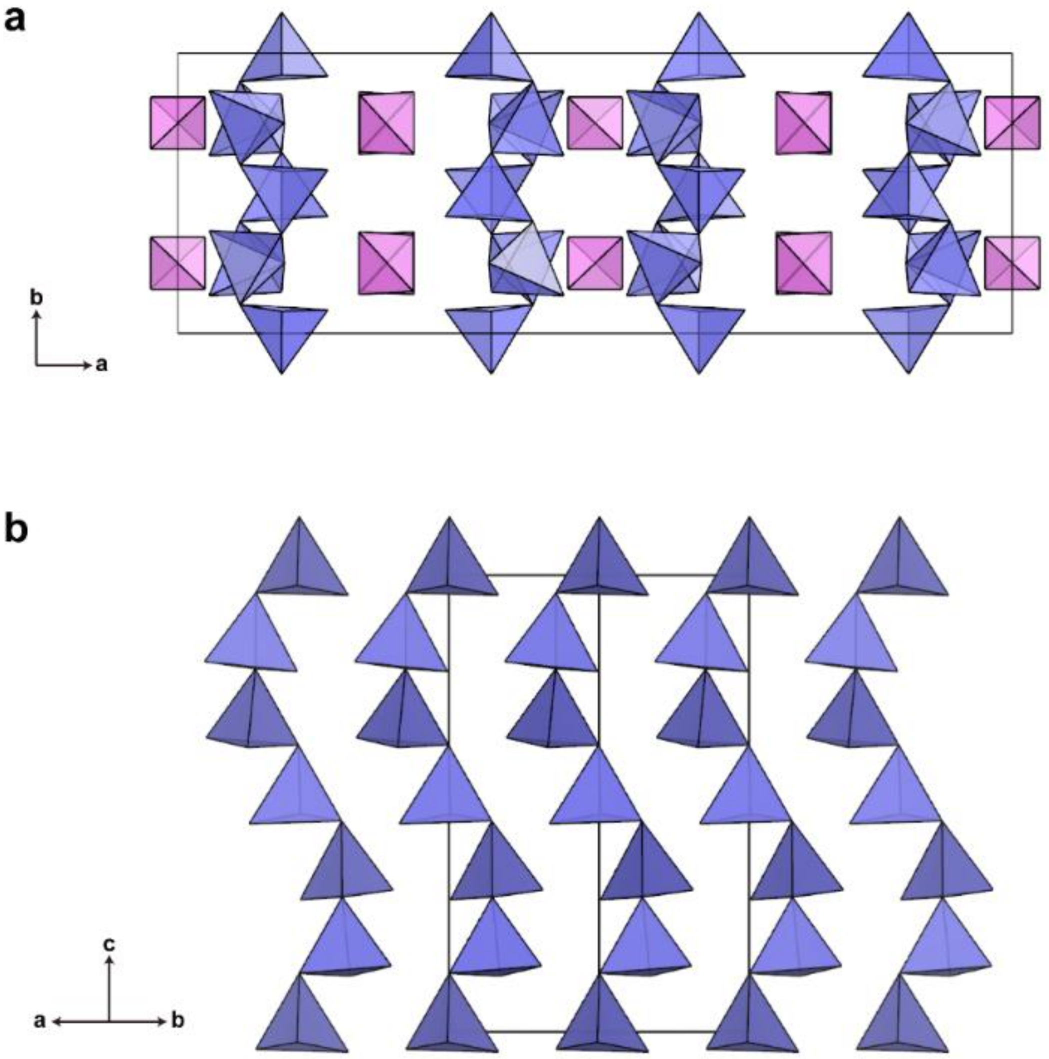


**Figure S9**. Crystal structure frameworks of (a) $Li_{21}Ge_8P_3S_{34}$ and (b) $Li_2GeS_3$, highlighting only the $GeS_4$ (violet) and $PS_4$ (pink) tetrahedra. The structures are viewed along the [001] and [110] directions, respectively.

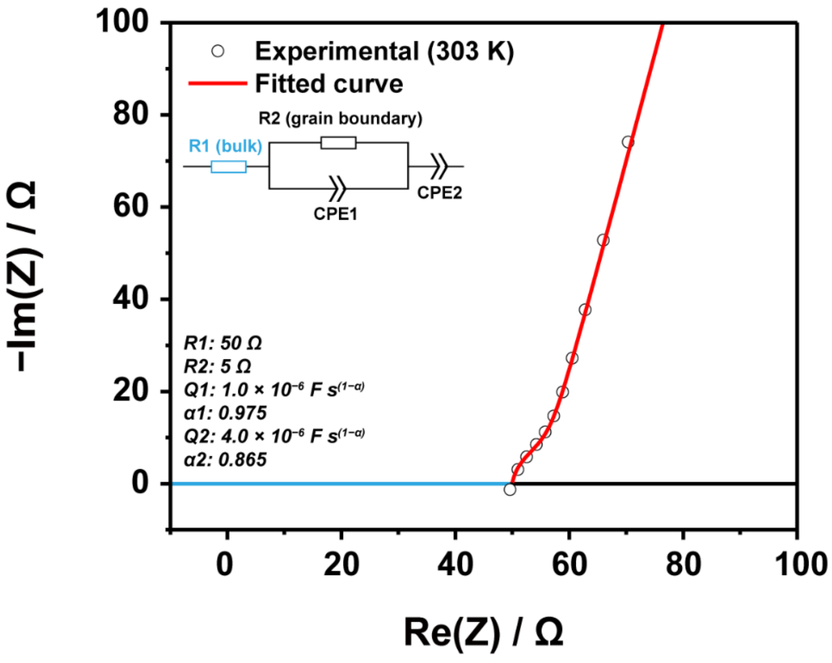


**Figure S10**. Nyquist plots for the $Li_{21}Ge_8P_3S_{34}$ at 303 K. Insets in each figure depict the corresponding equivalent circuit model, with circuit component values indicated at the bottom of the respective figures. Solid red lines represent the fitted curves, while experimental data points are shown as circles. The bulk resistance in the equivalent circuit model is highlighted in sky blue.

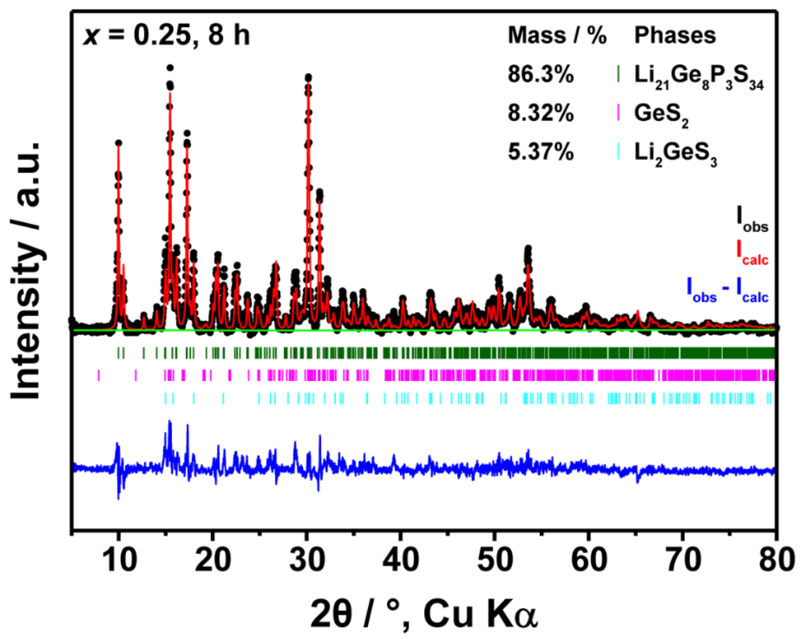


**Figure S11**. Powder X-ray Rietveld refinement profile for $x$ = 0.25 in $Li_{2-x}Ge_{1-x}P_xS_3$ (heat-treated for 8 h), with $GeS_2$ and $Li_2GeS_3$ impurities marked by pink and cyan Bragg reflection bars, respectively.

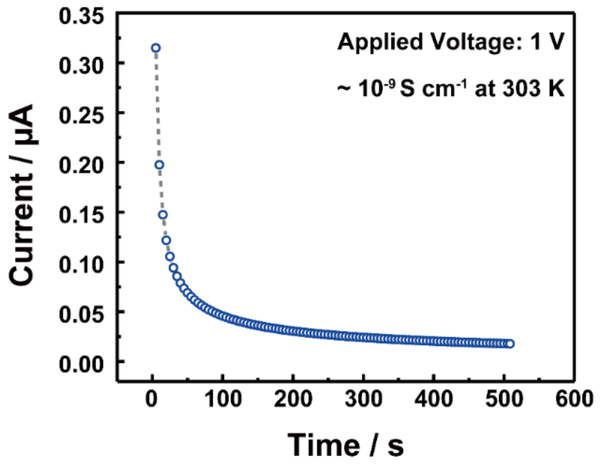


**Figure S12**. Current-time curves of the SS/$Li_{21}Ge_8P_3S_{34}$/SS press cell under DC polarization at 1 V.

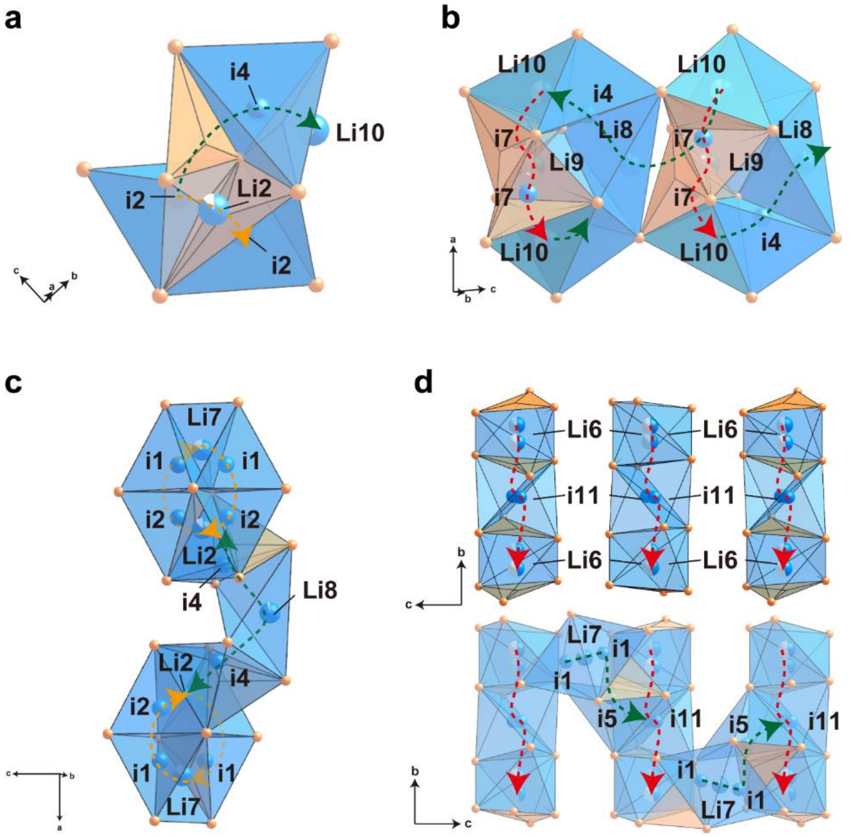


**Figure S13**. The lithium substructures of $Li_{21}Ge_8P_3S_{34}$ along lithium-ion diffusion pathways, determined from the bond valence energy landscape. It highlights (1) face-shared tetrahedral connectivity: (a) [i2–i4–Li10] and [i2–Li2–i2], (b) [Li10–i7–Li9–i7–Li10] and [Li10–i7–Li8–i4–Li10], (c) [i1–Li7–i1–i2–Li2–i2–i1] and [i2–i4–Li8–i4–i2]; and (2) face-shared octahedral connectivity: (d) [Li6–i11–Li6], with its diffusion network linked via face-shared tetrahedral connectivity [i1–Li7–i1–i5–i11]. The orange region corresponds to the saddle point area, indicated as “s#” in **Table S6**.

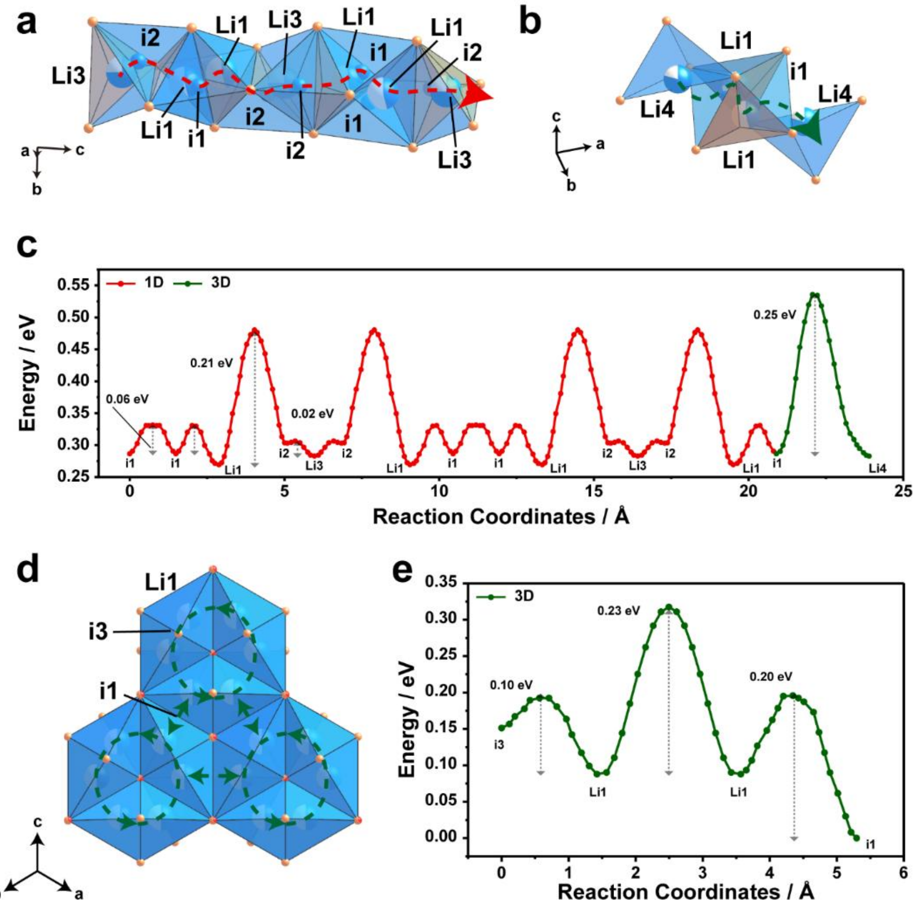


**Figure S14**. (a, b) Lithium substructures of $Li_{10}GeP_2S_{12}$ (ICSD #188887) within the lithium-ion diffusion pathways shown in (c). (d) Lithium substructure of $Li_6PS_5Cl$ (ICSD #259205) within the lithium-ion diffusion pathways shown in (e).[17,18]

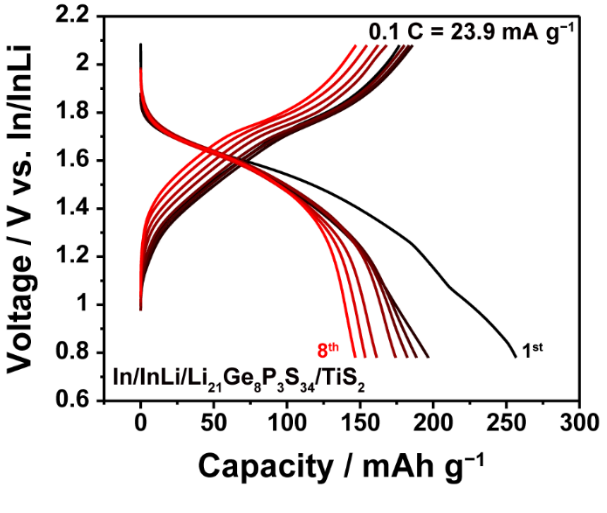


**Figure S15**. Galvanostatic discharge–charge profile of the In/InLi/$Li_{21}Ge_8P_3S_{34}$/$TiS_2$ press cell configuration at a rate of 23.9 mA $g^{-1}$ over eight cycles.

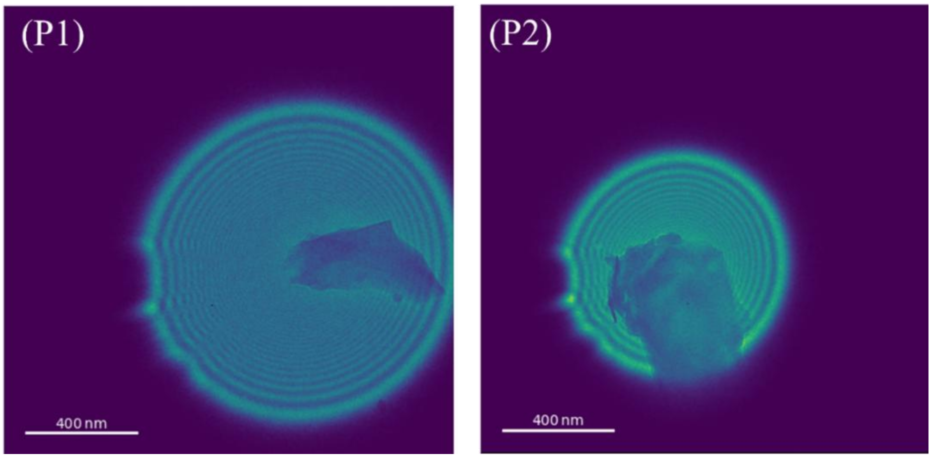


**Figure S16**. P1 and P2 particles exposed to the electron beam during 3D ED analysis. The dose rate and total dose used for data acquisition were 0.54 $e^{-}Å^{-2}s^{-1}$ and 70 $e^{-}Å^{-2}$ for P1 and 1.03 $e^{-}Å^{-2}s^{-1}$ and 133 $e^{-}Å^{-2}$ for P2, respectively.

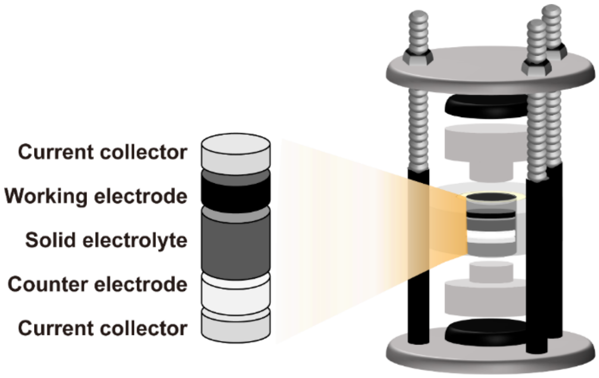


**Figure S17**. Schematic of the press cell configuration used for electrochemical performance measurements.

**Table S1.** Ionic conductivity of the nominal compositions in the $Li_{2-x}Ge_{1-x}P_xS_3$ ($0.1 \le x \le 0.4$, heat-treated for 8 h) substitution series.[a]

| nominal compositions (*x*) | total ionic conductivity (mS cm$^{-1}$ at 303 K) |
|---|---|
| 0.1 | $6.1(1) \times 10^{-3}$ |
| 0.25 | 0.92(2) |
| 0.333 | 0.55(1) |
| 0.4 | $3.1(1) \times 10^{-2}$ |

[a] Error range was calculated based on the pellet lengths with three measurements.

**Table S2.** Crystallographic data, atomic coordinates, site occupancies, and isotropic thermal displacement parameters for $Li_{21}Ge_8P_3S_{34}$, obtained from dynamical refinement of 3D electron diffraction (3D ED) data.

| | |
|---|---|
| Crystal system | orthorhombic |
| Formula weight (g mol$^{-1}$) | 1893.2 |
| Space group, Z | *Ccce* (origin choice 1) (no.68), 4 |
| Lattice parameters, volume | *a* = 35.4318 Å, *b* = 11.9008 Å, *c* = 12.6105 Å |
| | *V* = 5317.446 Å$^3$ |
| Density$_{calc}$ (g/cm$^3$) | 2.3648 |
| Temperature (K) | 298 |

| Atoms | *x* | *y* | *z* | Wyckoff | Occupancy | $U_{iso}$ (Å$^2$) |
|---|---|---|---|---|---|---|
| Ge1 | −0.08824(8) | −0.4765(3) | −0.1384(2) | 16*i* | 1.0 | 0.0118(6) |
| Ge2 | −0.12751(8) | −0.7124(3) | −0.2827(2) | 16*i* | 1.0 | 0.0130(6) |
| S1 | −0.1270(2) | −0.5 | 0 | 8*e* | 1.0 | 0.0150(14) |
| S2 | −0.12409(13) | −0.1741(4) | −0.0496(3) | 16*i* | 1.0 | 0.0143(10) |
| S3 | −0.07392(12) | −0.6485(5) | −0.2000(3) | 16*i* | 1.0 | 0.0164(11) |
| S4 | −0.03786(13) | −0.3805(4) | −0.1110(3) | 16*i* | 1.0 | 0.0148(11) |
| S5 | −0.12482(14) | −0.3981(4) | −0.2620(3) | 16*i* | 1.0 | 0.0155(10) |
| S6 | −0.21774(13) | −0.3885(4) | −0.0887(3) | 16*i* | 1.0 | 0.0162(11) |
| S7 | −0.18087(12) | −0.6671(5) | −0.2098(3) | 16*i* | 1.0 | 0.0183(11) |
| S8 | −0.28174(14) | −0.5978(4) | −0.0972(3) | 16*i* | 1.0 | 0.0227(12) |
| S9 | −0.0326(2) | −0.0947(6) | 0.0975(3) | 16*i* | 1.0 | 0.0381(15) |
| P1 | −0.2494(2) | −0.5 | 0 | 8*e* | 1.0 | 0.018(2) |
| P2 | 0 | 0 | 0 | 4*a* | 1.0 | 0.020(2) |
| Li1 | 0 | −0.5 | 0 | 4*b* | 0.72(12) | 0.06(4) |
| Li2 | −0.0630(7) | −0.253(2) | 0.0152(14) | 16*i* | 0.99(6) | 0.028(9) |
| Li3 | −0.1905(6) | −0.477(2) | −0.2465(14) | 16*i* | 0.83(5) | 0.009(7) |
| Li4 | −0.1741(8) | −0.297(3) | 0.0260(2) | 16*i* | 0.92(6) | 0.046(11) |
| Li5 | −0.25 | −0.75 | −0.270(3) | 8*h* | 0.99(10) | 0.09(2) |
| Li6 | −0.0874(14) | 0 | 0 | 8*e* | 0.92(9) | 0.07(2) |
| Li7 | −0.3326(14) | −0.5 | 0 | 8*e* | 0.80(7) | 0.06(2) |
| Li8 | 0 | 0 | 0.241(3) | 8*g* | 0.78(9) | 0.04(2) |

**Table S3.** Selected interatomic distances (Å) and bond angles (°) in $Li_{21}Ge_8P_3S_{34}$.

| Ge coordination | Distance | | Angles |
|---|---|---|---|
| Ge1–S1 | 2.138(6) | S1–Ge1–S2 | 110.6(3) |
| Ge1–S2 | 2.238(6) | S1–Ge1–S3 | 112.2(2) |
| Ge1–S3 | 2.242(7) | S2–Ge1–S3 | 105.6(2) |
| Ge1–S6 | 2.251(6) | S4–Ge2–S5 | 113.4(3) |
| Ge2–S2 | 2.275(7) | | |
| Ge2–S3 | 2.268(4) | | |
| Ge2–S4 | 2.172(6) | | |
| Ge2–S5 | 2.113(6) | | |
| **P coordination** | | | |
| P1–S7 | 2.044(6) | S7–P1–S7 | 111.7(4) |
| P1–S8 | 2.074(6) | S7–P1–S8 | 112.5(3) |
| P2–S9 | 2.006(6) | | 104.2(2) |
| | | S8–P1–S8 | 111.9(4) |
| | | S9–P2–S9 | 113.5(3) |
| | | | 105.2(4) |
| | | | 109.8(4) |
| **Li coordination** | | | |
| Li1–S1 | 2.445(16) | S1–Li1–S2 | 111.2(9) |
| Li1–S2 | 2.557(17) | S1–Li1–S9 | 124.8(9) |
| Li1–S4 | 2.554(16) | S1–Li1–S4 | 111.2(10) |
| Li1–S9 | 2.385(16) | S2–Li1–S9 | 92.8(9) |
| Li2–S4 | 2.569(14) | S2–Li1–S4 | 110.7(8) |
| Li2–S7 | 2.538(17) | S4–Li1–S9 | 104.5(9) |
| Li3–S4 | 2.63(6) | S4–Li2–S4 | 109.6(13) |
| Li3–S5 | 2.28(6) | S4–Li2–S7 | 131.6(5) |
| Li3–S6 | 2.63(6) | S7–Li2–S7 | 88.4(12) |
| Li3–S8 | 2.37(6) | S4–Li3–S5 | 140(4) |
| Li4–S3 | 2.578(17) | S4–Li3–S6 | 99(4) |
| Li4–S5 | 2.353(14) | S4–Li3–S8 | 103(3) |
| Li4–S7 | 2.464(15) | S5–Li3–S6 | 107(3) |
| Li4–S8 | 2.349(15) | S5–Li3–S8 | 111(4) |
| Li5–S1 | 2.363(6) | S6–Li3–S8 | 81(3) |
| Li6–S1 | 2.73(3) | S3–Li4–S5 | 105.0(9) |
| | 2.55(3) | S3–Li4–S7 | 98.0(8) |
| Li6–S9 | 2.49(3) | S3–Li4–S8 | 108.7(9) |
| | 2.62(3) | S5–Li4–S7 | 123.9(10) |
| Li7–S4 | 2.619(17) | S5–Li4–S8 | 109.2(9) |
| Li7–S9 | 2.39(2) | S7–Li4–S8 | 110.5(9) |
| Li8–S8 | 2.73(2) | S1–Li5–S1 | 113.6(6) |
| Li9–S7 | 2.192(17) | S1–Li6–S1 | 91.2(12) |
| Li9–S8 | 2.62(4) | S1–Li6–S2 | 81.7(11) |
| Li10–S4 | 2.41(2) | | 105.5(13) |
| Li10–S5 | 2.61(3) | S1–Li6–S9 | 81.6(11) |
| Li10–S7 | 2.61(3) | | 94.9(14) |
| Li10–S8 | 2.32(3) | S2–Li6–S9 | 99.4(13) |

| | |
|---|---|
| | 128.8(15) |
| S9–Li6–S9 | 77.5(11) |
| S4–Li7–S4 | 112.9(16) |
| S4–Li7–S9 | 130.0(6) |
| S9–Li7–S9 | 84.3(13) |
| S5–Li8–S5 | 148.7(17) |
| S5–Li8–S7 | 86.8(8) |
| | 71.4(7) |
| S5–Li8–S8 | 91.7(5) |
| | 109.7(6) |
| S7–Li8–S7 | 91.8(12) |
| S7–Li8–S8 | 87.0(4) |
| | 178.3(9) |
| S8–Li8–S8 | 94.2(13) |
| S7–Li9–S7 | 107(5) |
| S7–Li9–S8 | 86.0(11) |
| | 120.0(15) |
| S8–Li9–S8 | 137(7) |
| S4–Li10–S5 | 122(5) |
| S4–Li10–S7 | 91.0(2) |
| S4–Li10–S8 | 119(5) |
| S5–Li10–S7 | 80(3) |
| S5–Li10–S8 | 119(4) |
| S7–Li10–S8 | 107(4) |

**Table S4.** Bond valence sums (BVSs) and the number of counted bond valences (BVs) for $Li_{21}Ge_8P_3S_{34}$, calculated using softBV (v1.2.7).[12]

| Atom | oxidation state | number of counted BVs | BVS (v.u.) |
|---|---|---|---|
| Ge1 | +4 | 4 | 4.01 |
| Ge2 | +4 | 4 | 4.10 |
| S1 | –2 | 5 | –1.88 |
| S2 | –2 | 4 | –2.15 |
| S3 | –2 | 3 | –2.18 |
| S4 | –2 | 6 | –1.85 |
| S5 | –2 | 5 | –1.96 |
| S6 | –2 | 4 | –2.21 |
| S7 | –2 | 6 | –2.04 |
| S8 | –2 | 6 | –2.00 |
| S9 | –2 | 5 | –2.02 |
| P1 | +5 | 4 | 4.87 |
| P2 | +5 | 4 | 5.28 |
| Li1 | +1 | 4 | 1.01 |
| Li2 | +1 | 4 | 0.94 |
| Li3 | +1 | 4 | 1.08 |
| Li4 | +1 | 4 | 1.08 |
| Li5 | +1 | 4 | 1.15 |
| Li6 | +1 | 5 | 0.95 |
| Li7 | +1 | 4 | 1.00 |
| Li8 | +1 | 6 | 0.82 |
| Li9 | +1 | 4 | 1.18 |
| Li10 | +1 | 4 | 1.05 |

**Table S5.** Results from the polyhedral template matching algorithm used to analyze the framework of $Li_{21}Ge_8P_3S_{34}$. For comparison, $Li_{10}GeP_2S_{12}$ and *β*-, *α*-$Li_3PS_4$ were also analyzed to ensure consistency with existing literature.

| crystal structure | sulfur sublattices | literature |
|---|---|---|
| $Li_{21}Ge_8P_3S_{34}$ | face-centered cubic (*fcc*): 41.18%, hexagonal close-packed (*hcp*): 47.06%, and body-centered cu bic (*bcc*): 11.76% | - |
| $Li_{10}GeP_2S_{12}$[a] | *bcc*: 100% | *bcc*[b] |
| *β*-$Li_3PS_4$[c] | *hcp*: 100% | *hcp*: 100%[d] |
| *α*-$Li_3PS_4$[e] | *hcp*: 24.92%, *bcc*: 75.08% | *hcp*: 25%, *bcc*: 75%[f] |

a ref. [17]
b ref. [19]
c ref. [20]
d, e, f ref. [21]

**Table S6.** Computed Li positions, saddle points, and interstitial site energies in $Li_{21}Ge_8P_3S_{34}$, calculated using softBV along the main pathways.[12] Note that the listed Li positions correspond to equivalent sites, as shown in **Table 1**.

| site | multiplicity | *x* | *y* | *z* | site energy (eV)* |
|---|---|---|---|---|---|
| Li10 | 16 | 0.178 | 0.525 | 0.222 | 0.029 |
| i1 | 16 | 0.097 | 0.767 | 0.819 | 0.100 |
| Li6 | 16 | 0.508 | 0.275 | 0.493 | 0.105 |
| s2 | 8 | 0 | 0.25 | 0.507 | 0.111 |
| Li8 | 8 | 0.25 | 0 | 0.465 | 0.120 |
| Li7 | 8 | 0.089 | 0.25 | 0.25 | 0.128 |
| s3 | 16 | 0.589 | 0.25 | 0.771 | 0.135 |
| i2 | 16 | 0.153 | 0.225 | 0.319 | 0.136 |
| Li2 | 8 | 0.161 | 0.25 | 0.25 | 0.180 |
| i4 | 16 | 0.7 | 0.933 | 0.333 | 0.182 |
| s5 | 16 | 0.161 | 0.242 | 0.271 | 0.184 |
| i5 | 16 | 0.553 | 0.092 | 0.847 | 0.191 |
| i7 | 16 | 0.225 | 0.958 | 0.326 | 0.206 |

| | | | | | |
|---|---|---|---|---|---|
| Li9 | 8 | 0.25 | 0.5 | 0.236 | 0.227 |
| s7 | 8 | 0 | 0.5 | 0 | 0.264 |
| s8 | 16 | 0.072 | 0.183 | 0.139 | 0.276 |
| s9 | 16 | 0.189 | 0.033 | 0.215 | 0.281 |
| s15 | 16 | 0.181 | 0.183 | 0.146 | 0.312 |
| s16 | 16 | 0.239 | 0.517 | 0.201 | 0.324 |
| s19 | 16 | 0.206 | 0.533 | 0.194 | 0.340 |
| s22 | 16 | 0.492 | 0.892 | 0.007 | 0.357 |
| s23 | 16 | 0.125 | 0.275 | 0.181 | 0.366 |
| s24 | 16 | 0.264 | 0.975 | 0.882 | 0.366 |
| s26 | 16 | 0.525 | 0.067 | 0.924 | 0.373 |
| s29 | 16 | 0.231 | 0.458 | 0.417 | 0.382 |

*Note that site energies do not correspond to the activation energy. The activation energy can be calculated as the difference between these site energies along the Li diffusion pathways.

**Table S7.** Unit cell parameters for the particles P1 and P2, along with refined data obtained from powder X-ray and neutron diffraction analyses.

| Data | Unit cell parameters |
|---|---|
| P1[a] | $a$ = 34.94 Å, $b$ = 11.87 Å, $c$ = 12.44 Å, $\alpha$ = 90.38°, $\beta$ = 90.90°, and $\gamma$ = 90.84° |
| P2 | $a$ = 34.93 Å, $b$ = 11.69 Å, $c$ = 12.41 Å, $\alpha$ = 90.29°, $\beta$ = 90.81°, and $\gamma$ = 89.53° |
| Powder X-ray/neutron diffraction | $a$ = 35.4145(6) Å, $b$ = 11.8907(2) Å, $c$ = 12.6004(2) Å, $\alpha$ = 90.00°, $\beta$ = 90.00°, and $\gamma$ = 90.00° |

[a] Optical distortions were later refined based on the unit cell refined for powder diffraction data. 1.25% error was observed for calibration constant used in electron diffraction.

**Table S8.** Data processing details for the *Ccce* space group of P1.

| **Data collection and reduction** | | | |
|---|---|---|---|
| Rotation Range (°) | 67 | Laue class | mmm |
| Resolution (Å) | 0.8 | $R_{meas}$ (obs) (%) | 12.8 |
| Completeness (%) | 80.3 | $R_{int}$ (obs) (%) | 10.2 |
| I/σ | 3.1 | No. of Observed reflections | 1028 |
| $CC_{1/2}$ (%) | 98.1 | No. of all reflections | 2445 |
| **Structure Refinement - Kinematical** | | **Structure Refinement - Dynamical** | |
| $R_{obs}$ (%) | 24.26 | $R_{obs}$ (%) | 11.31 |
| $wR_{obs}$ (%) | 34.52 | $wR_{obs}$ (%) | 11.24 |
| Goodness of fit | 3.54 | Goodness of fit | 3.68 |

## REFERENCES

[1] S. Hori, M. Kato, K. Suzuki, M. Hirayama, Y. Kato, R. Kanno, *J. Am. Ceram. Soc.* **2015**, *98*, 3352–3360.

[2] A. Huq, M. Kirkham, P. F. Peterson, J. P. Hodges, P. S. Whitfield, K. Page, T. Hűgle, E. B. Iverson, A. Parizzi, G. Rennich, *J. Appl. Crystallogr.* **2019**, *52*, 1189–1201.

[3] L. Palatinus, P. Brázda, M. Jelínek, J. Hrdá, G. Steciuk, M. Klementová, *Acta Crystallogr. Sect. B* **2019**, *75*, 512–522.

[4] G. M. Sheldrick, *Acta Crystallogr. Sect. A* **2015**, *71*, 3–8.

[5] V. Petříček, L. Palatinus, J. Plášil, M. Dušek, *Z. für Krist. - Cryst. Mater.* **2023**, *238*, 271–282.

[6] S. Gholam, J. Hadermann, *Ultramicroscopy* **2024**, 114022.

[7] P. Brázda, M. Klementová, Y. Krysiak, L. Palatinus, *IUCrJ.* **2022**, *9*, 735–755.

[8] B. H. Toby, R. B. V. Dreele, *J. Appl. Crystallogr.* **2013**, *46*, 544–549.

[9] P. W. Betteridge, J. R. Carruthers, R. I. Cooper, K. Prout, D. J. Watkin, *J. Appl. Crystallogr.* **2003**, *36*, 1487–1487.

[10] L. Palatinus, G. Chapuis, *J. Appl. Crystallogr.* **2007**, *40*, 786–790.

[11] M. Hušák, B. Kratochvíl, *J. Appl. Crystallogr.* **2003**, *36*, 1104–1104.

[12] H. Chen, L. L. Wong, S. Adams, *Acta Crystallogr. Sect. B Struct. Sci. Cryst. Eng. Mater.* **2019**, *75*, 18–33.

[13] K. Momma, F. Izumi, *J. Appl. Crystallogr.* **2011**, *44*, 1272–1276.

[14] A. Stukowski, *Model. Simul. Mater. Sci. Eng.* **2010**, *18*, 015012.

[15] P. M. Larsen, S. Schmidt, J. Schiøtz, *Model. Simul. Mater. Sci. Eng.* **2016**, *24*, 055007.

[16] M. C. Burla, R. Caliandro, B. Carrozzini, G. L. Cascarano, C. Cuocci, C. Giacovazzo, M. Mallamo, A. Mazzone, G. Polidori, *J. Appl. Crystallogr.* **2015**, *48*, 306–309.

[17] A. Kuhn, J. Köhler, B. V. Lotsch, *Phys. Chem. Chem. Phys.* **2013**, *15*, 11620–11622.

[18] M. A. Kraft, S. P. Culver, M. Calderon, F. Böcher, T. Krauskopf, A. Senyshyn, C. Dietrich, A. Zevalkink, J. Janek, W. G. Zeier, *J. Am. Chem. Soc.* **2017**, *139*, 10909–10918.

[19] Y. Wang, W. D. Richards, S. P. Ong, L. J. Miara, J. C. Kim, Y. Mo, G. Ceder, *Nat. Mater.* **2015**, *14*, 1026–1031.

[20] R. Mercier, J. -P. Malugani, B. Fahys, G. Robert, J. Douglade, *Acta Crystallogr. Sect. B* **1982**, *38*, 1887–1890.

[21] K. Kaup, L. Zhou, A. Huq, L. F. Nazar, *J. Mater. Chem. A* **2020**, *8*, 12446–12456.
